# Resident alveolar macrophages are highly susceptible to the induction of RIPK1/RIPK3-mediated necroptosis which promote efficient pulmonary immune response

**DOI:** 10.1038/s41419-025-08372-8

**Published:** 2025-12-22

**Authors:** Takumi Adachi, Kazufumi Matsushita, Koubun Yasuda, Masakiyo Nakahira, Mai Imasaka, Michihiko Sugimoto, Zhuohao Yang, Kouji Kobiyama, Tomoya Hayashi, Ken J. Ishii, Yasuo Yoshioka, Masaki Ohmuraya, Yoshitaka Shirasaki, Etsushi Kuroda

**Affiliations:** 1 Department of Immunology, Hyogo Medical University School of Medicine, Nishinomiya, Hyogo Japan; 2 Department of Genetics, Hyogo Medical University School of Medicine, Nishinomiya, Hyogo Japan; 3 Research Center for Advanced Science and Technology, The University of Tokyo, Meguro-ku, Tokyo Japan; 4 Division of Vaccine Science, Department of Microbiology and Immunology, The Institute of Medical Science, The University of Tokyo, Minato-ku, Tokyo Japan; 5 Vaccine Creation Group, Research Institute for Microbial Diseases, The University of Osaka, Suita, Osaka Japan; 6 The Research Foundation for Microbial Diseases of The University of Osaka, Suita, Osaka Japan

**Keywords:** Immune cell death, Adjuvants, Cell death and immune response, Alveolar macrophages, Mucosal immunology

## Abstract

Alveolar macrophages (AMs) play a crucial role in protecting the lungs from pathogens by inducing immunogenic cell death (ICD). However, the type of cell death that effectively induces protective immunity remains to be fully understood. In our investigation of the mechanisms regulating AM activation and lung immune responses, we found that AMs are highly susceptible to necroptosis, a form of ICD. Treatment with pan-caspase inhibitors, such as emricasan or benzyloxycarbonyl-Val-Ala-Asp(OMe)-fluoromethyl ketone (ZVAD-fmk), directly induced cell death in AMs, resulting in the release of interleukin (IL)-1α in the lungs. This phenomenon was not observed in mouse lung fibroblasts or bone marrow-derived macrophages, indicating a cell type-specific sensitivity. The process was mediated by receptor-interacting protein kinase (RIPK)1 and RIPK3, and notably occurred without any additional necroptosis triggers such as tumor necrosis factor (TNF) or second mitochondria-derived activator of caspase (SMAC) mimetics. Moreover, activation of the RIPK1–RIPK3 signaling pathway not only triggered necroptosis but also promoted IL-1α production and release. These responses were absent in AMs lacking functional RIPK1 kinase activity or deficient in RIPK3. Importantly, RIPK1/RIPK3-mediated cell death and IL-1α release were sufficient to trigger lung immune responses, as shown by increased antigen-specific IgG and IgA production, which was significantly decreased in mice deficient in necroptosis or lacking the IL-1 receptor. Taken together, these findings demonstrate that the pan-caspase inhibitor emricasan induces necroptotic cell death in AMs and may act as a promising AM-targeted adjuvant to enhance lung-specific acquired immunity.

## Introduction

Alveolar macrophages (AMs) serve as sentinels that clear microorganisms they encounter and environmental particulate matter inhaled into the alveoli, and are involved in the resolution of inflammation and tissue repair [1, 2]. AMs also have the potential to induce robust inflammatory responses and produce proinflammatory cytokines such as interleukin (IL)-1α and tumor necrosis factor (TNF) [3, 4], resulting in the promotion of effective adaptive immunity [3, 5].

Immunogenic cell death (ICD), which includes necrosis, pyroptosis, autophagic cell death, and necroptosis, is defined as a highly proinflammatory mode of cell death and accelerates inflammatory responses, resulting in effective induction of acquired immunity [6]. Necroptosis, a type of programmed cell death (or regulated cell death) with necrotic features, is initiated by various signaling molecules, including TNF receptors (TNFRs), Toll-like receptors (TLRs), and Z-DNA-binding protein 1 (ZBP1) [7]. Canonical receptor-interacting protein kinase 1 (RIPK1)/RIPK3-mediated necroptosis has been well documented in reports analyzing TNF-TNFR1 engagement in the cell death pathway [8, 9]. Holler et al. found that RIPK1 is responsible for programmed necrotic cell death [10]. RIPK1 activates RIPK3-mediated mixed lineage kinase domain-like protein (MLKL) phosphorylation and execution of necroptosis [11, 12], resulting in the release of intracellular inflammatory molecules called damage-associated molecular patterns (DAMPs), including high mobility group box 1 (HMGB-1) and IL-1α [13]. Caspase-8 is the initiator caspase of extrinsic apoptosis; it is triggered by the engagement of death receptors and their ligands [14] and is involved in maintaining cellular homeostasis by directly cleaving RIPK1 to inhibit necroptosis [14–17]. Therefore, genetic deletion of caspase-8 or suppression of its enzymatic activity by inhibitors induces necroptosis [9, 17, 18].

We have been focusing on pulmonary mucosal vaccination because pathogen-specific secretory IgA responses are reported to be more effective than those of other antibody classes in neutralizing or eliminating pathogens at the infection site [19, 20]. We previously demonstrated that AMs are the main source of IL-1α and play a critical role in the induction of adaptive immune responses in the lungs [3]. Based on the observation, we have been looking for a substance that induces cell death and release of IL-1α in the airways, which may act as an effective pulmonary adjuvant. However, despite being a potent inducer of IL-1α release from AMs, aluminum hydroxide (alum) is a persistent inorganic particle that cannot be used clinically as an intranasal mucosal adjuvant because it causes chronic inflammation when administered in the airways [3]. During our research on the development of pulmonary mucosal adjuvants, we unexpectedly discovered that the pan-caspase inhibitor induces necroptotic cell death coupled with robust release of IL-1α by inhibiting caspase activity in AMs. This effect was not observed in bone marrow-derived macrophages or lung fibroblasts, which explains the cell type-specific susceptibility to the ICD.

In this study, we demonstrated that AMs, but not bone marrow-derived macrophages (BMDMs) or lung fibroblasts, are uniquely susceptible to caspase inhibition-induced cell death and IL-1α release, a process that is entirely dependent on RIPK1/RIPK3. We also demonstrated that intranasal administration of a pan-caspase inhibitor emricasan with model antigen to mice induces systemic antigen-specific IgG in the serum and mucosal IgA in the bronchoalveolar lavage fluid (BALF). Furthermore, using *Ripk1*^DN/DN^, *Ripk3*^−/−^ and *Il1r1*^−/−^ mice, we found that the adjuvanticity of emricasan is entirely dependent on RIPK1/RIPK3-mediated necroptosis and largely dependent on necroptosis-driven IL-1 signaling. These results suggest that this unique susceptibility to the ICD in AMs contributes to the induction of protective immunity in the lungs.

## Materials/subjects and methods

### Animals and tissues

Female and male C57BL/6J and C57BL/6N mice (8–12 weeks of age) used in this study were purchased from Japan SLC (Shizuoka, Japan) or CLEA Japan (Tokyo, Japan). We thank Dr. Ken J. Ishii (The University of Tokyo, Japan) for providing *Il1r1*-deficient mice [21] and Dr. Manolis Pasparakis (University of Cologne, Germany) for providing *Ripk1*^D138N/D138N^ (*Ripk1*^DN/DN^) transgenic mice [22]. All mice used in this study were on a C57BL/6 J background except for *Ripk3*^−/−^ mice (C57BL/6 N) and were maintained under specific pathogen-free conditions.

### Reagents

All the reagents used in this study are listed in Supplemental Table.

### Preparation of gRNA/Cas9 complex to generate RIPK3-deficient mice

The gRNAs were designed using CRISPRdirect [23] and CRISPick [24, 25] to target promoter and intron 3 of the *Ripk3* gene. The gRNA sequences were as follows: *Ripk3*-1, 5’-AAGAGAGACTGGCTATCGTG-3’; *Ripk3*-2, 5’-TAATGCACCCTCACGGACCC-3’. The corresponding crRNA, tracrRNA, and Cas9 nuclease were purchased from Integrated DNA Technologies (IDT, Coralville, IA).

### Electroporation

Male and female C57BL/6 mice (CLEA Japan) were used as sperm and oocyte donors. Female mice were superovulated, and then zygotes were prepared by in vitro fertilization. They were cultured in KSOM medium (ARK Resource, Kumamoto, Japan) before and after electroporation. Electroporation was performed using a 1-mm gap electrode (CUY501P1-1.5) and a super electroporator NEPA21 (Nepa Gene, Chiba, Japan). Zygotes were washed with Opti-MEM (Thermo Fisher Scientific, Waltham, MA) and then placed in the electrode gap filled with 5 µL of Opti-MEM solution containing 200 ng/µL Cas9 protein and 6 pmol/µL gRNA (crRNA/tracrRNA complex). They were cultured in KSOM medium overnight, and surviving two-cell-stage embryos were transferred to the oviducts of pseudopregnant Jcl:ICR female mice (CLEA Japan).

### Intranasal administration of emricasan

Emricasan (0.2–0.8 mg/kg) was administered to the mice intranasally, and BALF was collected 6–12 h post-administration. IL-1α and TNF levels were measured using ELISA, as described below. Immune cells recruited to the BALF were stained with anti-CD11c-Brilliant Violet 421, SiglecF-PE, CD45-Pacific blue, CD11b-PE, Gr-1- and PerCP-Cy5.5, and propidium iodide (a viability dye) and then evaluated using a cell analyzer (SP6800; Sony Biotechnology, San Jose, CA).

### Immunization

Ovalbumin (OVA, 10 μg) or recombinant type A influenza virus strain H1N1 A/California/07/09 HA antigen (5 μg), kindly donated by Dr. Yoshioka (The University of Osaka, Japan), were administered to the airway of mice via intranasal route on day 0 and 10, with emricasan (0.8 mg/kg), alum (100 μg), rIL-1α (50 ng), rIL-1α (50 ng) with rTNF (100 ng) or DMSO (2% in PBS) as control group. The mice were sacrificed 7 days after the last administration, and serum and BALF were collected. OVA-specific IgA and IgG_1_ levels were measured using ELISA, as described below. Possible endotoxin contamination of OVA was tested using Endospecy® ES-50M and determined to be less than 0.1 EU/mg.

### Cell preparation and culture

Freshly isolated AMs were prepared from BALF by instilling ice-cold phosphate-buffered Saline (PBS; 1 mL × 3 times) into the lungs. In vitro-cultured alveolar macrophages were prepared with the method used in the previous study [3] with some modifications. Briefly, mice lungs were excised from mice and digested in 5 mL RPMI 1640 medium supplemented with DNase I (200 μg/mL) and collagenase type A (200 U/mL). Following mincing with sterile scissors, tissues were incubated at 37 °C for 1 h and mechanically dissociated using a gentleMACS™ Dissociator (program B.01; Miltenyi Biotech). Cell suspensions were washed twice in PBS, filtered through 40 µm preseparation filters (Miltenyi Biotech), and resuspended in DMEM supplemented with 10% FCS and insulin (5 µg/mL), without antibiotics. Cells were seeded in 75-cm² culture flasks (Corning, USA). After 6 to 7 days, CD11c^+^ Siglec F^+^ AM-like cells and the fibroblasts proliferated and differentiated. The macrophages were easily detached by reciprocal shaking (120 strokes/min) for 2 h at 37 °C, and the floating cells were collected as AM-like cells for in vitro experiments. Cells that remained vigorously attached to the surface of the flask were subcultured two to three times to remove residual macrophages and used as mouse lung fibroblasts. AM-like cells were further purified using biotin anti-mouse CD11c antibody, Streptavidin Microbeads and the MACS (Bergisch Gladbach, Germany) separation system to enrich CD11c+ cells. As previously reported, in vitro cultures of AMs enabled us to obtain a large number of cells (5.0 × 10^6^/T75 flask) (Fig. S1) [3]. Mouse lung fibroblasts were prepared in parallel from the lung cell culture. BMDMs were differentiated from bone marrow cells isolated from the femur and tibia of mice. The macrophages were grown in RPMI 1640 supplemented with 10% FCS, 1% penicillin/streptomycin, and 10 ng/mL recombinant mouse macrophage colony-stimulating factor (M-CSF). After incubation for 4 to 5 days, the cells were washed and detached using 0.25% trypsin/1 mM EDTA.

The obtained cells were cultured in 96-well plates in RPMI 1640 supplemented with 10% FCS, and were treated with emricasan, zVAD-fmk, or other ligands (2’3’-cGAMP, CpG ODN (K3), Poly(I:C), R848, Pam3CsK4, or LPS from *Escherichia coli* (O55:B5) or cultured with DMSO (0.1%) as a control. In some experiments, cells were pretreated with inhibitors (necrostatin-1 [1 or 10 μM] or GSK872 [1 or 10 μM]) prior to necroptosis induction. After stimulation, supernatants and cells were collected.

### Characterization of cells

AMs freshly isolated from BALF or cultured (as described above) were stained with anti-CD11c-Brilliant Violet 421, SiglecF-PE, CD45-Pacific blue, and propidium iodide and characterized using a cell analyzer (SP6800, Sony Biotechnology).

### LDH cytotoxicity assay

The cytotoxicity of emricasan and other reagents on macrophages was measured by lactose dehydrogenase (LDH) release. After the cells were exposed to the reagents, the cultured supernatants were collected, and LDH activity was detected with an LDH assay kit (Promega, MADISON, WI). The maximum LDH control was prepared by induction of necrotic cell death using either 1% Triton X-100 (which was contained in the kit), or by subjecting cells to a freeze-thaw cycle three times (control cell lysate). The control cell lysate was serially diluted to create a standard curve of OD values versus cell lysate concentrations. The maximum LDH value was set as 100% cytotoxicity, and the cytotoxicity (%) of each sample was calculated by plotting the OD value against the standard curve.

### Luminescence cell viability assay

Cultured AM were cultured in 96-well plates (1 × 10^5^ cells/well) and treated with emricasan (40 μM). After 1, 3, 6, and 12 h of incubation, the cell viability was measured using Celltiter Glo luminescent cell viability assay (Promega) according to the manufacturer’s protocol. Briefly, at the end of the stimulation, the cells were subjected to the Celltiter Glo assay system working solution for 15 min and the luminescence intensity was measured using a GloMax 96 microwell luminometer (Promega).

### ELISA (Enzyme-Linked Immunosorbent Assay)

IL-1α, TNF, and IL-12p40 levels were measured using an ELISA MAX™ Deluxe Set (BioLegend, San Diego, CA), and IL-6 levels were measured using a DuoSet ELISA (R&D Systems, Minneapolis, MN) according to the manufacturer’s protocols. Briefly, ELISA plates were coated with capture antibodies (indicated concentrations) diluted with PBS overnight at 4 °C. The wells were blocked with 1% BSA–PBS for 30 min. Then, samples and standards (recombinant cytokines) were added to the wells and incubated overnight at 4 °C. After washing with 0.05% Tween 20–PBS, biotin-labeled secondary antibodies were added to the wells and incubated for 1 h at room temperature. After washing with 0.05% Tween 20–PBS, HRP-conjugated streptavidin diluted with BSA 1% PBS were added to the wells and incubated for 30 min at room temperature. After washing with 0.05% Tween 20–PBS, A working solution of 3,3’,5,5’-tetramethylbenzidine (TMB) substrate was added to the wells after the washing step. Shortly after the reaction developed, it was stopped by adding 1 M H_2_SO_4_. Absorbance was read at 450 nm using a microplate reader. Serum and BALF OVA-specific IgA and IgG_1_ were measured by ELISA [26]. Briefly, ELISA plates were coated with OVA (10 μg/mL) in PBS overnight at 4 °C. The wells were blocked with 1% BSA–PBS for 30 minutes. Then, samples and standards were added to the wells and incubated overnight at 4 °C. After washing with 0.05% Tween 20–PBS, secondary antibodies (horseradish peroxidase [HRP]-conjugated anti-mouse IgG_1_ or IgA antibodies, 0.5 μg/mL) were added to the wells and incubated for 1 h at room temperature. A working solution of TMB substrate was added to the wells after the washing step. Shortly after the reaction developed, it was stopped by adding 1 M H_2_SO_4_. Absorbance was read at 450 nm using a microplate reader.

### LCI-S (live-cell imaging of secretion activity) analysis of IL-1α secretion from AMs

IL-1α secretion was imaged using LCI-S as described previously [27]. Briefly, time-resolved measurements were performed with an automated inverted ECLIPSE Ti2-E microscope (Nikon, Tokyo, Japan) equipped with a high numerical aperture objective lens (CFI Apo TIRF 60× oil, numerical aperture = 1.49; Nikon), a stage-top incubator INUBG2TF-WSKM (Tokai Hit, Shizuoka, Japan), and a scientific CMOS camera (ORCA-Fusion BT; Hamamatsu Photonics, Shizuoka, Japan). An LED (SOLA Light Engine, Lumencor, Beaverton, OR) and a 640-nm LD laser, LDI-NIR (89 North, Williston, VT) were the light sources. The following were the excitation (Ex) and emission (Em) filters and the dichroic mirror (DM) used for the TIRF illumination of IL-1α via LCI-S: a fluorescence filter cube (TRITC-A-Basic, Semrock IDEX Health & Science, West Henrietta, NY) for the epi-illumination of SYTOX Orange (Thermo Fisher Scientific); ZET405/470/555/640x (Ex; Chroma Technology, Bellows Falls, VT), ZT561dcrb-UF2 (Em; Chroma Technology), and ZT405/470/555/640rpc-UF1 (DM, Chroma Technology). Cells were suspended in medium, and 3000 cells were added to each chamber (5 mm in diameter) of a four-condition LCI-S chip (LCI-SPQ002; Live Cell Diagnosis, Saitama, Japan), where the anti-IL-1α antibody (mouse IL-1 alpha/IL-1F1 antibody; R&D Systems) was immobilized. Immediately before observation, the culture supernatant was replaced with fresh culture medium containing 100 nM SYTOX Orange and 30 nM biotinylated antibody against IL-1α (mouse IL-1 alpha/IL-1F1 biotinylated antibody; R&D Systems) coupled with CF660R-labeled streptavidin (Biotium, Fremont, CA) with or without 40 μM emricasan. Mineral oil (Merck, Darmstadt, Germany) was layered on the medium to prevent evaporation. A total of 16 fields of view were scanned in each chamber repetitively to detect the extracellular release of IL-1α and dead cell nuclei stained with SYTOX Orange continuously using LCI-S.

### Measurement of caspase-8 activity

Cells cultured in 96-well plates were treated with emricasan as described above. At the end of the stimulation, the cells were subjected to the Caspase-8 Glo assay system working solution for 15 min, and the luminescence intensity was measured using a GloMax 96 microwell luminometer (Promega).

### Western blot analysis

Cells were lysed with RIPA lysis buffer (50 mM Tris/HCl [pH 8.0], 150 mM NaCl, 1% NP-40 substitute, 0.1% SDS, and 0.5% sodium deoxycholate), including proteinase inhibitor cocktail. To denature the proteins, the lysate was mixed with SDS loading buffer (0.25 M Tris/HCl [pH 6.8], 8% SDS, 20% glycerol, 20% 2-mercaptoethanol, 0.008% bromophenol blue) and heated at 95 °C for 5 min. The lysates were subjected to glycine-SDS-PAGE (10% acrylamide separation gel) following transfer to PVDF membrane using an iBlot 2 Gel Transfer Device (Thermo Fisher). Immunoblotting was performed by probing with anti-caspase-8 (1/1000), anti-RIPK1 (1/1000), anti-RIPK3 (1/1000), and anti-ERK (1/1000) antibodies diluted with 5% skim milk in TBST (0.05% Tween 20, 10 mM Tris/HCl, pH 7.5). After a vigorous wash, the membranes were incubated with HRP-conjugated donkey anti-rabbit antibody (1/200) or HRP-conjugated goat anti-mouse antibody (1/2000) diluted with 5% skim milk in TBST. Enhanced chemiluminescence substrate was added after washing. Western blot signals were detected using an ImageQuant LAS 4010 (GE Healthcare Life Sciences, Marlborough, MA).

### Gene expression profiling by RNA-seq

Gene expression profiling of AMs treated with emricasan for 6 hours (or resting as a control) was performed using the Illumina HiSeq 2500 platform (Illumina, San Diego, CA). Please see the acknowledgment section below for all methods.

### RT-PCR (qPCR)

Total RNA was purified from cells using a Fastgene RNA basic kit (NIPPON Genetic, Tokyo, Japan), and cDNA was synthesized using ReverTra Ace qPCR RT Mix (Toyobo, Osaka, Japan). Quantitative polymerase chain reaction was performed using premix Ex Taq, SYBR Green premix Ex Taq (Takara, Shiga, Japan), TaqMan Gene Expression Assays (Applied Biosystems), and a Thermal Cycler Dice Real-time System 2 (Takara). The target gene expression was normalized to the expression of the housekeeping gene *Actb*. The primers [28] and probes (Applied Biosystems, Waltham, MA) used in this study are listed in the key resources table.

### Statistics

Statistical analysis was performed using Prism 8 software (GraphPad Software, Boston, MA). Results are shown as mean ± standard deviation. Student’s *t*-test (unpaired, two-tailed) was used for comparison of two groups. Tukey’s multiple comparisons test (for ordinary one-way ANOVA) and Kruskal–Wallis test (for nonparametric test) were used for multiple comparison of three or more groups. *P* values are shown as *p < 0.05, **p < 0.01, and ***p < 0.001.

## Results

### Emricasan, a pan-caspase inhibitor, induces cell death and robust IL-1α production in AMs

**Fig. 1:**
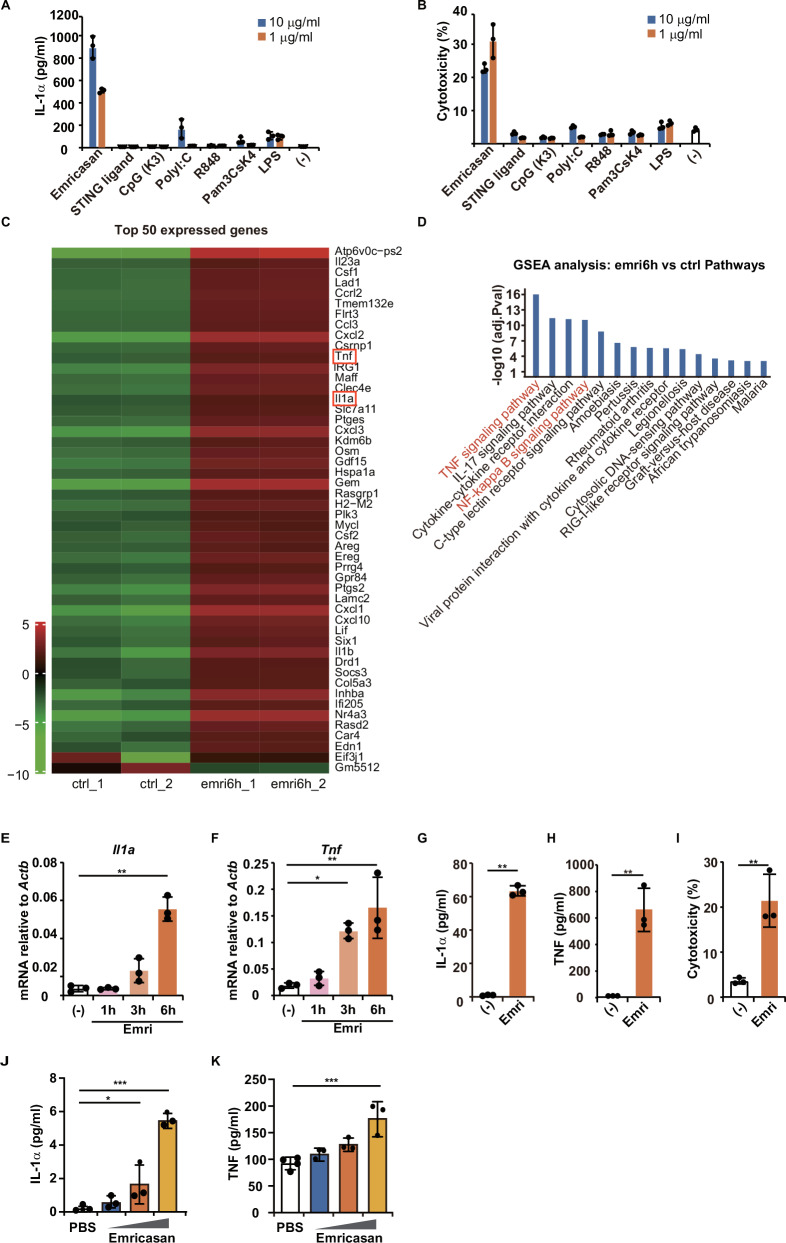
Emricasan, a pan-caspase inhibitor, induces cell death and robust IL-1α production in alveolar macrophages (AMs) and in the respiratory tracts of mice. **A**, **B** AMs were cultured with or without emricasan, STING ligand (2’ 3’ -cGAMP), CpG (K3), poly I:C, R848, Pam3CsK4, and LPS (1 or 10 μg/mL) for 12 h (*n* = 3/group). The culture supernatants were collected and IL-1α was measured using ELISA (A) and cytotoxicity with LDH cytotoxicity assays (**B**). **C**, **D** Bulk RNA sequencing of alveolar macrophages cultured with emricasan (40 μM) for 6 h. The top 50 differentially expressed genes from alveolar macrophages treated with emricasan for 6 h (emri6h) vs. untreated (ctrl) cells are shown in the heatmap (**C**). Gene set enrichment analysis (GSEA) of differentially expressed genes in AMs treated with emricasan (emri6h) relative to untreated cells (ctrl) (**D**). **E**, **F** Primary AMs isolated from C57BL/6 mice were cultured in the presence of emricasan (40 μM) for 1, 3, and 6 h (*n* = 3/group). The levels of *Il1a* (**E**) and *Tnf* (**F**) mRNA expression were measured by qPCR. **G**–**I** Primary AMs isolated from C57BL/6 mice were cultured with or without emricasan (Emri; 40 μM) for 12 h (*n* = 3/group). Cytokine levels (IL-1α and TNF) in the culture supernatant were measured using ELISA (**G**, **H**). Cytotoxicity was measured with LDH assays (**I**). **J**, **K** C57BL/6 mice were administered serially diluted emricasan (0.2–0.8 kg/mL) intranasally (*n* = 3–4/group). Ten hours after administration, BALF was collected by instilling 500 μL of PBS. Levels of the cytokines IL-1α (**J**) and TNF (**K**) in BALF were measured using ELISA. Data were representative of at least two independent experiments. Error bars represent mean ± SD. *P* values were calculated with one-way ANOVA (Tukey’s multiple comparisons test or the Kruskal–Wallis test. *P < 0.05, **P < 0.01, and ***P < 0.001, ns (not significant).

Previously, we identified that AMs constitutively express IL-1α, which contributes to the activation of lung immune responses [3]. To evaluate potential pulmonary adjuvants, we screened candidate molecules focusing on IL-1α production from cultured AMs. Among the candidate adjuvant molecules tested in cultured AMs, including TLRs ligands (lipopolysaccharides [LPS], PAM3CSK4, R848, Poly[I:C], and CpG ODN), STING ligand (2’3’-cGAMP) [29] and emricasan, the most effective IL-1α production and cell death were induced by emricasan, a pan-caspase inhibitor (Fig. 1A, B).

We also evaluated other proinflammatory cytokines released from the cultured AMs stimulated with these adjuvants. We found that emricasan is a unique compound with the ability to induce robust IL-1α production, accompanied by cellular injury and moderate TNF release, but it did not induce the release of the typical proinflammatory cytokines IL-6 and IL-12p40 (Fig. S2A–C). By contrast, TLRs and STING ligands induced robust TNF, IL-6, and IL-12p40 release, but did not induce cell death or IL-1α release from cultured AMs (Fig. S2A–C and Fig. 1A, B). We confirmed that another well-known pan-caspase inhibitor, zVAD-fmk, also induced IL-1α production and cell death (Fig. S3A, B). We then directly compared alveolar macrophages from C57BL/6 J and C57BL/6 N mice, as well as from male and female C57BL/6 J mice, with respect to cell death induction and IL-1α release. No obvious differences were observed between these strains or sexes (Fig. S4).

To understand the characteristics of inflammatory responses induced by emricasan, we performed a comprehensive bulk RNA-seq analysis of cultured AMs treated with emricasan for 6 h (Fig. 1C, D). The top 50 most expressed genes are listed in Fig. 1C, showing that *Tnf*, *Il1a*, and *Il1b* are proinflammatory cytokines selectively and robustly expressed in cultured AMs treated with emricasan. We also used gene set enrichment analysis (GSEA) based on the Kyoto Encyclopedia of Genes and Genomes [30] to characterize emricasan-induced cell death in cultured AMs (Fig. 1D). GSEA analysis revealed that the TNF and NF-kB signaling pathways were highly activated by emricasan treatment, and that these pathways may be involved in emricasan-induced cell death in cultured AMs (Fig. 1D). These RNA-seq results indicate that emricasan induced de novo synthesis of IL-1α and TNF in addition to the release of IL-1α extracellularly; this was confirmed by qPCR results (Fig. 1E, F). We showed that primary AMs isolated from the BALF of mice also released IL-1α and TNF and cell death was induced after treatment with emricasan (Fig. 1G–I).

Intriguingly, intranasal administration of emricasan to C57BL/6 mice demonstrated that a single dose induced significant levels of IL-1α and TNF in the BALF after 6 hours in a dose-dependent manner (Fig. 1J, K).

### Emricasan induces cell death and robust IL-1α production by stimulating canonical RIPK1/RIPK3-mediated necroptosis in AMs

**Fig. 2:**
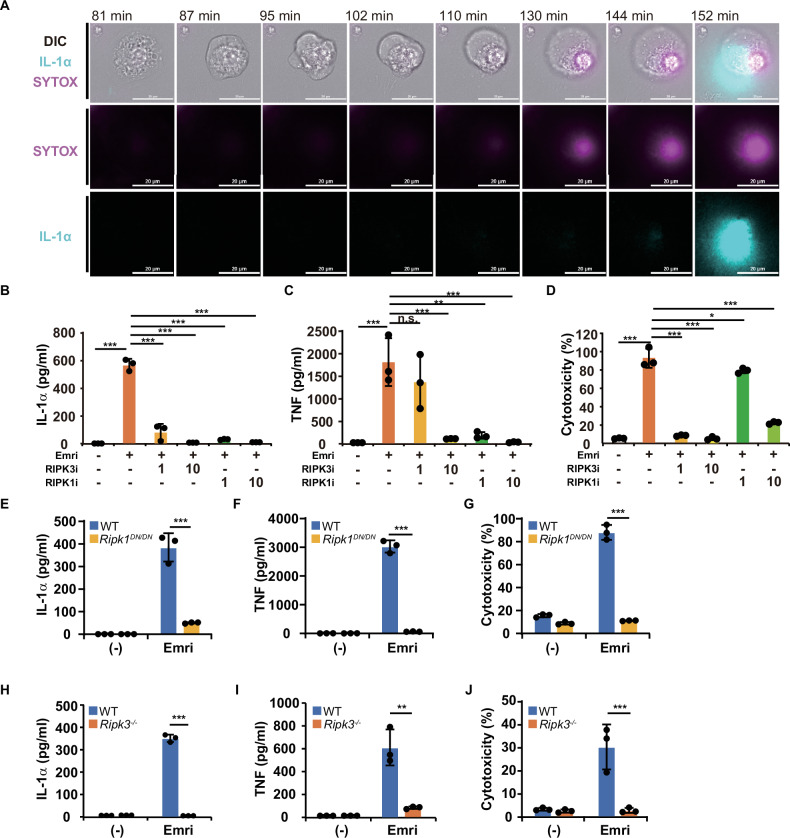
Emricasan induces cell death and robust IL-1α production by inducing canonical RIPK1/RIPK3-mediated necroptosis in alveolar macrophages (AMs). **A** Representative LCI-S live-cell images of cultured AMs isolated from the lungs of C57BL/6 mice and treated with emricasan (40 μM). Combined or independent images from a differential interference contrast microscope (DIC); IL-1α signal (cyan) and SYTOX (magenta) are shown. Scale bar: 20 μm. **B**–**D** Cultured AMs isolated from the lungs of C57BL/6 mice were pretreated with RIPK1 or RIPK3 inhibitors (RIPK1i and RIPK3i, respectively) at the indicated concentration (1 or 10 μM) for 1 h and then cultured with emricasan (Emri, 40 μM) for 12 h (*n* = 3/group). Cytokine levels (IL-1α and TNF) in the culture supernatant were measured using ELISA (**B**, **C**). Cytotoxicity was measured with LDH assays (**D**). **E**–**J** Cultured AMs isolated from the lungs of C57BL/6 WT, *Ripk1*^DN/DN^ and *Ripk3*^−/−^ mice were cultured with emricasan (Emri, 40 μM) for 12 h (*n* = 3/group). IL-1α and TNF levels in the culture supernatant were measured using ELISA (**E**, **H**: IL-1α; **F**, **I**: TNF). Cytotoxicity was measured with LDH assays (**G**, **J**). Data were representative of at least two independent experiments. Error bars represent mean ± SD. *P* values were calculated with the Kruskal–Wallis test or one-way ANOVA (Tukey’s multiple comparisons test). *P < 0.05, **P < 0.01, and ***P < 0.001, ns (not significant).

To investigate the type of cell death, we first performed live-cell imaging of secretion activity (LCI-S), which can visualize cell death (or loss of plasma membrane integrity) and the timing of IL-1α release simultaneously [31]. As shown in Fig. 2A and supplementary video (Video S1), increase in SYTOX uptake was observed shortly after the apparent swelling and bubbling of the cellular membrane, followed by an increase in cellular permeability resulting in IL-1α release, which may be a characteristic of necrosis or necroptosis [32]. We also conducted kinetic experiments at 3-, 6-, and 12-h post-stimulation. Cell death became evident at 6 h post-stimulation, at which point the release of IL-1α and TNF was detected (Fig. S5).

We hypothesized that the release of IL-1α from cultured AMs treated with emricasan is associated with necroptotic cell death. To prove this hypothesis, we used selective inhibitors of the endogenous molecules responsible for necroptotic cell death pathways. Cultured AMs pretreated with necrostatin-1 (RIPK1 inhibitor) or GSK872 (RIPK3 inhibitor) and then treated with emricasan had significantly reduced production of IL-1α and TNF, and less cytotoxicity in a dose-dependent manner (Fig. 2B–D). We confirmed these results by comparing cultured AMs from WT mice and RIPK1 dominant-negative mutant (*Ripk1*^DN/DN^) mice or RIPK3 knockout (*Ripk3*^−/−^) mice which was newly established in this study and confirmed the deletion of RIPK3 at the protein level (Fig. S6). The results clearly showed that emricasan treatment of cultured AMs from these genetically modified mice failed to induce IL-1α and TNF production or cytotoxicity (Fig. 2E–J). We also provide direct evidence from LCI-S analysis showing that the release of IL-1α is a consequence of cell death, and that the cytotoxicity following treatment with emricasan or zVAD-fmk is highly regulated and occurs only through RIPK3-associated necroptosis (Fig. S7). These results suggested that emricasan induces canonical RIPK1/RIPK3-mediated necroptosis in AMs, leading to their robust IL-1α production.

### AMs are a cell type highly susceptible to the induction of necroptosis

**Fig. 3:**
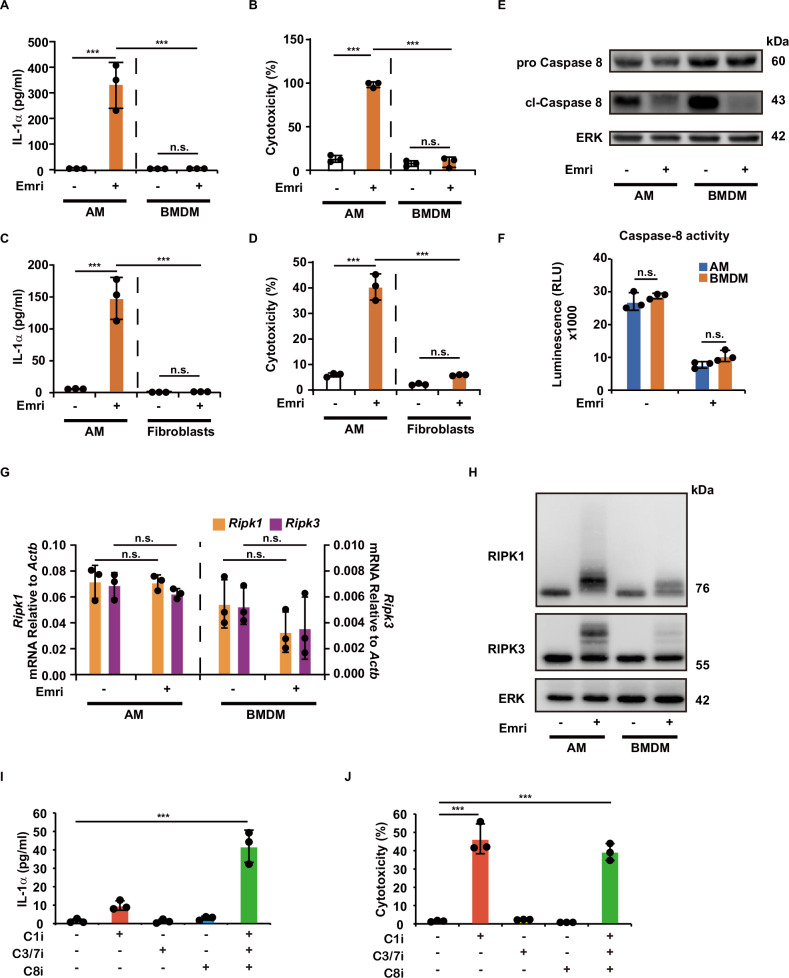
AMs are a cell type highly susceptible to the induction of necroptosis. **A**–**D** Cultured AMs, bone marrow-derived macrophages (BMDMs) or lung fibroblasts isolated from C57BL/6 mice were cultured for 12 h with emricasan (Emri, 40 μM) (*n* = 3/group). IL-1α levels in the culture supernatant were measured using ELISA (**A**, **C**), and cytotoxicity was measured with LDH assays (**B**, **D**). **E**, **F** The effects of emricasan on caspase-8 were assessed in cultured AMs and BMDMs. Western blot analysis of caspase-8, cleaved caspase-8, and ERK protein levels in cultured AMs and BMDMs treated with emricasan (40 μM) for 6 h (**E**). Cells treated with emricasan (40 μM) for 5 h were subjected to Caspase-8-Glo assay (**F**) (*n* = 3/group). **G**, **H** The effects of emricasan on RIPK1 and RIPK3 expression in cultured AMs and BMDMs were assessed by qPCR and western blot analysis. RIPK1 and RIPK3 mRNA expression was assessed by qPCR after culturing AMs and BMDMs that were treated with emricasan for 6 h (**G**) (*n* = 3/group). RIPK1, RIPK3, and ERK protein levels were analyzed by western blot analysis (**H**). **I**, **J** In vitro-cultured alveolar macrophages isolated from the lungs of C57BL/6 mice were cultured in the presence or absent of caspase inhibitors (C1i caspase-1 inhibitor, C3/7i caspase-3/7 inhibitor, C8i caspase-8 inhibitor, 40 μM) for 12 h. IL-1α level of the culture supernatant was measured by ELISA (**I**). The cytotoxicity was measured by LDH assay (**J**). Data were representative of at least two independent experiments. Error bars represent mean ± SD. *P* values were calculated by the Kruskal–Wallis test or one-way ANOVA analysis of variance (Tukey’s multiple comparisons test). *P < 0.05, **P < 0.01, and ***P < 0.001, ns (not significant).

Previous studies showed that induction of necroptosis by pan-caspase inhibitors in BMDMs requires priming with LPS or TNF [8, 33]. However, on the basis of our results, AMs are highly susceptible to necroptosis without any priming. To assess cell type-specific sensitivity to the induction of necroptosis, we compared cultured AMs and M-CSF-derived BMDMs or mouse lung fibroblasts after treatment with emricasan. The results clearly showed robust production of IL-1α and cell death in cultured AMs, but not in BMDMs (Fig. 3A, B) or mouse lung fibroblasts (Fig. 3C, D) following treatment.

We then focused on the signal events upstream of RIPK1/RIPK3 activation in the necroptotic cell death. It has been reported that induction of RIPK1-dependent necroptosis typically requires death-domain-related signaling, such as the signal events downstream of TNF-TNFR1 engagement [22]. To clarify the contribution of TNF signaling in these responses, we used cultured AMs from *Tnf*^−/−^ mice and compared IL-1α release and cell death with those of littermate mice. The results revealed no statistically significant difference in the release of IL-1α or cell death between cultured AMs from *Tnf* ^−/−^ and those from *Tnf* ^+/−^ or *Tnf* ^+/+^ mice, suggesting a minimal contribution of TNF-TNFR1 signaling. Alternatively, other death-domain receptor-related ligands may contribute to emricasan-induced necroptosis in cultured AMs (Fig. S8).

Caspase-8 serves as a critical regulator of RIPK1 activity under homeostatic conditions by cleaving RIPK1 to prevent the execution of necroptosis [17]. Furthermore, a previous study reported that caspase-8 is required for M-CSF-dependent differentiation of macrophage precursor without inducing apoptosis [34]. In line with these observations, we detected the mature form of caspase-8 (cleaved caspase-8) in cultured AM and BMDMs lysates in the steady-state condition by western blot analysis (Fig. 3E). The result also clearly showed that the level of cleaved caspase-8 decreased in lysates of cultured AMs and BMDMs treated with emricasan (Fig. 3E). Parallel to that, remarkable caspase-8 activity was detected in both AMs and BMDMs, which was impaired by the treatment of emricasan (Fig. 3F). These results show no significant difference in caspase-8 expression and enzymatic function between AMs and BMDMs before and after the treatment of emricasan.

We then compared the gene expression levels of RIPK1 and RIPK3, which are the key molecules in necroptosis downstream of caspase-8 inhibition, in these macrophages by qPCR and found that neither type of macrophage upregulated these genes, and the levels were comparable following treatment with emricasan (Fig. 3G). However, western blot analysis revealed that the accumulation of polyubiquitinated- or phosphorylated-like high molecular weight ladders of RIPK1 and RIPK3, which were remarkable in cultured AMs, was not evident in BMDMs (Fig. 3H).

We then tried to clarify whether the mechanism of emricasan-induced necrosis-like cell death was dependent on caspase-8 inhibition using the caspase-8-specific inhibitor benzyloxycarbonyl (Cbz)-Ile-Glu (OMe)-Thr-Asp (OMe)-FMK (Z-IETD-FMK). Notably, Z-IETD-FMK alone could not induce necroptosis-like cell death (Fig. 3I, J). Therefore, we hypothesized that emricasan would exhibit greater inhibitory efficacy against caspase-8 enzymatic activity than Z-IETD-FMK, and we evaluated this by performing a luminescence-based caspase-8 activity assay. The results demonstrated that the inhibitory potency of emricasan against caspase-8 was not significantly different from that of Z-IETD-FMK (Fig. S9A). We also used a caspase-1 selective inhibitor, VX-765 (belnacasan), a caspase-3/7 selective inhibitor, N-Ac-Asp-Glu-Val-Asp-CHO (Ac-DEVD-CHO), and a mixture of these and found that simultaneous inhibition with these caspases significantly upregulated IL-1α release and cell death (Fig. 3I, J). Furthermore, we found that caspase-1 inhibition by VX-765 induced necroptosis-independent cell death, which was not blocked by necrostatin-1 or GSK872 treatment. However, the mixture of caspase-1 -3/7 and -8 inhibitors shifted the death to necroptosis, suggesting that the necroptosis-like cell death required a multiple caspases inhibition rather than a single inhibition of caspases (Fig. S9B).

Collectively, these results demonstrate that AMs are susceptible to RIPK1/RIPK3-mediated necroptosis, and that multiple caspase inhibition is required for the induction of cell death and IL-1α release.

### Emricasan functions as a pulmonary mucosal adjuvant that induces necroptosis upon airway administration

**Fig. 4:**
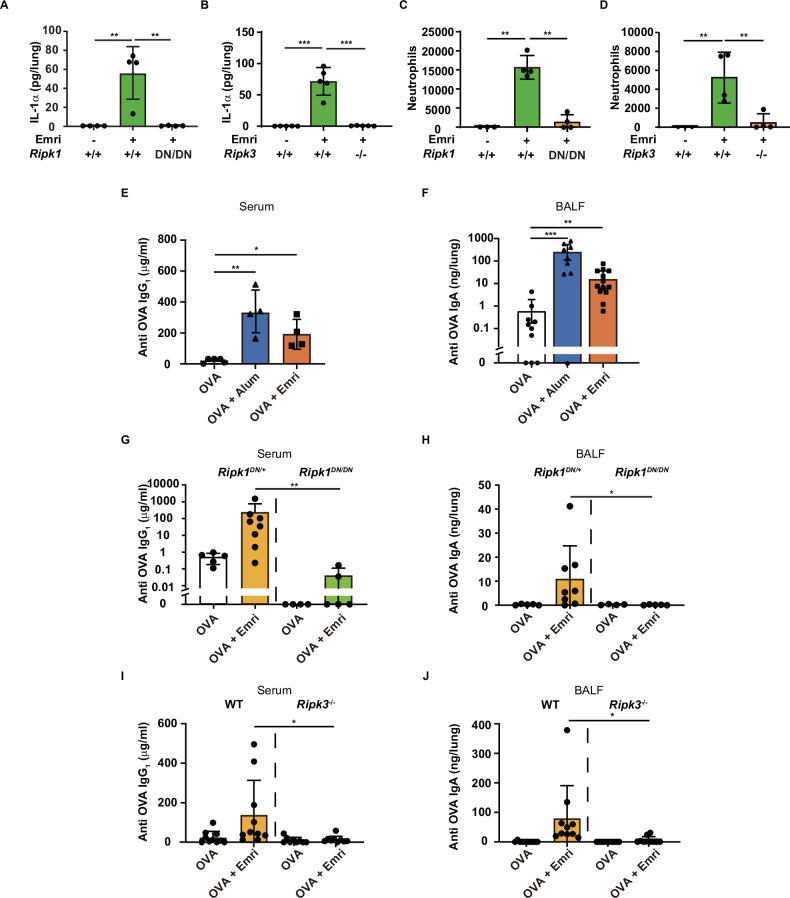
Emricasan is a mucosal adjuvant that induces necroptosis when administered intranasally. **A**–**D** C57BL/6 WT, *Ripk1*^DN/DN^ and *Ripk3*^−/−^ mice received intranasal emricasan (0.8 kg/mL) in PBS. After 10 h, BALF was collected by instilling PBS 500 μL. IL-1α levels in BALF were measured using ELISA (**A**, **B**) (*n* = 4–5/group). Neutrophil infiltration into BALF was quantified using a cell analyzer 12 h after emricasan administration (**C**, **D**) (*n* = 3–4/group). The gating strategy for evaluating immune cells in the BALF post-administration is shown in Figs. S11, S12. **E**, **F** C57BL/6 WT mice received OVA (10 μg) with or without aluminum hydroxide (alum, 3.3 mg/kg) or emricasan (Emri, 0.8 mg/kg) intranasally on days 0 and 10. On day 17, the mice were sacrificed and serum and BALF were collected. OVA-specific IgG_1_ (**E**) (*n* = 4–5/group) and IgA (**F**) (*n* = 9–13/group) titers were analyzed using ELISA. (G-J) C57BL/6 WT and *Ripk1*^DN/DN^ or *Ripk3*^−/−^ mice received OVA (10 μg) with or without emricasan (Emri, 0.8 mg/kg) intranasally on days 0 and 10. On day 17, the mice were sacrificed and serum and BALF were collected (**G**, **H**: *n* = 4–8/group, **I**, **J**: *n* = 9–10/group). OVA-specific IgG_1_ (**G**, **I**) and IgA (**H**, **J**) titers were analyzed using ELISA. Data were representative of two independent experiments (**A**–**E**) or are pooled from two independent experiments (**F**–**J**). Error bars represent mean ± SD. *P* values were calculated with the Kruskal–Wallis test or one-way ANOVA (Tukey’s multiple comparisons test). *P < 0.05, **P < 0.01, and ***P < 0.001, ns (not significant).

To clarify the contribution of necroptosis to IL-1α production in a mouse model of respiratory inflammation, we analyzed immune responses in the lungs of WT, *Ripk1*^DN/DN^, or *Ripk3*^−/−^ mice following airway administration of emricasan. As we expected, 6 hours after emricasan administration, IL-1α production in the lungs was significantly reduced in the genetically modified mice (Fig. 4A, B). In parallel with IL-1α levels, infiltration of neutrophils into the lungs was also significantly reduced in genetically modified mice 12 h after emricasan administration (Fig. 4C, D). These results suggest that emricasan induces necroptosis of AMs in the lungs in the same manner as observed in the in vitro study, and that the released IL-1α may function as a pulmonary adjuvant. To assess the potential of emricasan as a pulmonary adjuvant, OVA was administered to the airway of mice via the intranasal route, either alone, in combination with emricasan, or with alum on days 0 and 10. Seven days after the final administration (day 17 of the course), serum and BALF were collected. As shown in Fig. 4E, the group of mice treated with intranasal emricasan showed significant induction of OVA-specific serum IgG_1_, comparable to that induced by alum. Emricasan also significantly elevated OVA-specific IgA levels in BALF (Fig. 4F). To investigate the contribution of necroptosis to antibody responses, we evaluated the adjuvant effect of emricasan using *Ripk1*^DN/DN^ and *Ripk3*^−/−^ mice. The results showed that OVA-specific serum IgG_1_ and BALF IgA were significantly reduced in *Ripk1*^DN/DN^ (Fig. 4G, H) and *Ripk3*^−/−^ mice (Fig. 4I, J), demonstrating that the adjuvanticity of emricasan is almost entirely dependent on RIPK1–RIPK3-mediated necroptosis.

### The adjuvanticity of emricasan is mainly dependent on necroptosis-mediated IL-1 release

**Fig. 5:**
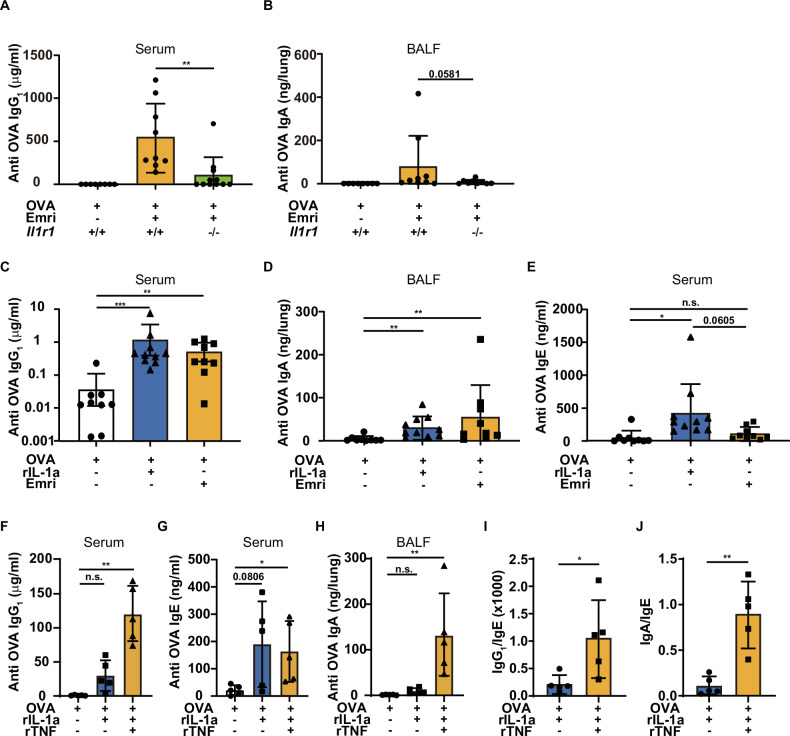
The adjuvanticity of emricasan is mainly dependent on necroptosis-mediated IL-1 release but has the unique property of reducing IgE production. **A**, **B** C57BL/6 *Il1r1*^+/−^ and *Il1r1*^−/−^ mice received OVA (10 μg) with or without emricasan (Emri, 0.8 mg/kg) intranasally on days 0 and 10. On day 17, the mice were sacrificed and serum and BALF were collected (*n* = 8–11/group). OVA-specific IgG_1_ (**A**) and IgA (**B**) titers were analyzed using ELISA. **C**–**E** C57BL/6 WT mice received OVA (10 μg) with or without rIL-1α (50 ng) or emricasan (Emri, 0.8 mg/kg) intranasally on days 0 and 10. On day 17, the mice were sacrificed and serum and BALF were collected (*n* = 9–10/group). OVA-specific IgG_1_ (**C**), IgA (**D**), and IgE (**E**) titers were analyzed using ELISA. **F**–**J** C57BL/6 WT mice received OVA alone, OVA (10 μg) with IL-1α (50 ng), or OVA (10 μg) with IL-1α (50 ng) with rTNF (100 ng) intranasally on days 0 and 10. On day 17, the mice were sacrificed and serum and BALF were collected (*n* = 5/group). Serum levels of OVA-specific IgG_1_ (**F**), IgE (**G**), and BALF levels of OVA-specific IgA (**H**) titers were analyzed using ELISA. The ratios of OVA-specific IgG_1_/IgE (**I**) and IgA/IgE (**J**) were calculated after the levels of each isotype were determined. Data were pooled from two independent experiments (**A**–**E**) or are representative of two independent experiments (**F**–**J**). Error bars represent mean ± SD. *P* values were calculated with the Kruskal–Wallis test or one-way ANOVA (Tukey’s multiple comparisons test). *P < 0.05, **P < 0.01, and ***P < 0.001, ns (not significant).

Next, we investigated whether IL-1 release through necroptosis contributes to antibody responses, using IL-1R1-deficient mice. The results showed that both OVA-specific serum IgG_1_ and BALF IgA were significantly reduced in these mice, indicating that the adjuvanticity of emricasan is largely dependent on necroptosis-mediated IL-1 release (Fig. 5A, B). These results suggest that IL-1α, a DAMP released during necroptotic cell death, contributes to the adjuvanticity of emricasan as an effective pulmonary mucosal adjuvant. To confirm the potential of IL-1α as a pulmonary mucosal adjuvant, we performed a side-by-side comparison of antibody responses in mice administered intranasal emricasan with those in mice administered recombinant IL-1α (rIL-1α). The results showed that the levels of OVA-specific serum IgG_1_ and BALF IgA induced by emricasan were comparable to those induced by rIL-1α (Fig. 5C, D). However, the results also showed that OVA-specific IgE production, which was significantly upregulated in the group of mice administered rIL-1α, was not significantly induced in the group that received emricasan (Fig. 5E). Because TNF was upregulated in AMs in response to emricasan (Fig. S2A), we therefore wondered whether emricasan-induced TNF production contributes to the regulation of an isotype shift toward IgE. To confirm the effect of TNF on the isotype shift, we compared the antibody responses of mice that received rIL-1α intranasally with or without recombinant TNF (rTNF). The results showed that co-administration of rIL-1α and rTNF was more effective in upregulating OVA-specific serum IgG_1_ and BALF IgA than rIL-1α alone, but did not significantly change OVA-specific serum IgE (Fig. 5F–H). Furthermore, we observed that the ratios of IgG_1_/IgE and IgA/IgE were significantly upregulated by the co-administration of rIL-1α with rTNF compared with rIL-1α alone (Fig. 5I, J). These findings may explain the role of TNF in regulating the isotype shift toward IgE. These results also suggest that emricasan is a potent candidate pulmonary mucosal vaccine adjuvant, promoting antigen-specific IgA in the airway mucosa and systemic IgG via RIPK1/RIPK3-mediated necroptosis of AMs in the lung, while reducing IgE production and inducing no ICD except in AMs. Finally, we conducted an immunization model using the human-applicable type A influenza virus strain H1N1 A/California/07/09 HA antigen to assess the efficacy of emricasan as a mucosal adjuvant. The results demonstrated that the intranasal administration of HA antigen with emricasan significantly increased HA-specific serum IgG_1_ and BALF IgA titers compared with administration of HA antigen alone (Fig. S10). Collectively, these findings indicate that emricasan functions as an intranasal mucosal adjuvant, stimulating systemic IgG and mucosal IgA production and thereby potentially enhancing host defense against respiratory pathogens such as influenza virus.

## Discussion

Necroptosis is a type of programmed (or regulated) cell death that is thought to function as an early defense mechanism against viral infections [35–37]. Although the importance of rapid inflammatory responses during the early phase of infection, particularly in airway immune defense, has been demonstrated in many studies, no reports have yet directly demonstrated the relationship between necroptosis and the induction of adaptive immune responses, such as IgA production. We have now demonstrated that RIPK1/RIPK3-mediated necroptosis is associated with antigen-specific IgG and IgA antibody responses.

To begin with, we found that in AMs, cell death was specifically induced by pan-caspase inhibitors without any additional necroptosis triggers such as TNF or SMAC mimetics compounds targeting cellular inhibitor of apoptosis proteins (cIAPs) or X-linked inhibitor of apoptosis protein (XIAP)s [11, 38]. We further demonstrated that caspase-8, an apoptosis initiator that regulates necroptosis and pyroptosis [10, 39, 40] and is involved in the differentiation and function of macrophages [34, 41–43], is expressed as a mature form under steady-state condition in AMs. Furthermore, in AMs, robust induction of necroptosis and IL-1α production required the inhibition of multiple caspases, including caspase-1, -3, -7, and -8. Based on these results, we raised a new pharmacological and biological question regarding the induction of necroptosis, although the detailed mechanisms remain to be clarified. In addition, we demonstrated that the cell death induced by pan-caspase inhibitors was almost entirely dependent on RIPK1/RIPK3-mediated necroptosis, which is generally associated with signaling downstream of TNFRs or related death-domain-containing receptors. However, we ruled out the involvement of the TNF-TNFR signaling axis in this process, and the precise upstream mechanisms also remain under investigation.

Next, we demonstrated that IL-1α release after induction of RIPK1/RIPK3-mediated necroptosis is responsible for the induction of antibody responses because mice lacking IL-1R1 had severely inhibited antibody responses. In fact, some studies have already shown that recombinant IL-1α has the potential to be an intranasal mucosal adjuvant [44, 45]; however, we observed characteristic differences in antibody responses, such as IgE production, when comparing recombinant IL-1α and emricasan. Hence, we speculate that, in addition to IL-1α, other signaling pathway such as death receptor-related signaling may also contribute to necroptosis-dependent antibody responses. We also found that emricasan stimulate AMs to release TNF. Previous studies reporting serological analysis of inflammatory bowel disease and rheumatoid arthritis patients who received infliximab, an anti-TNF drug, suggested that infliximab attenuates the immunogenicity of vaccines in these patients [46, 47]. In addition, in a mouse model of intranasal vaccination, co-administration of antigen with a highly bioactive modified recombinant TNF significantly upregulated both systemic and mucosal immune responses [48]. These studies indicated the immunogenicity of TNF for vaccinations. Our findings revealed that TNF contributes to isotype switching, suggesting that TNF may serve as a key factor in modulating the IgE response by emricasan.

No nasal adjuvants are approved for use in humans, and therefore we are interested in exploring and developing effective and clinically acceptable pulmonary mucosal adjuvants. In this study, we selected emricasan from a large number of caspase inhibitors because of its effectiveness and advantages for human use [49–51]. Emricasan, developed by Idun Pharmaceutical, is an irreversible pan-caspase inhibitor is currently under investigation for the treatment of nonalcoholic steatohepatitis (NASH) [50, 51], a condition characterized by extensive hepatocyte apoptosis. Unfortunately, emricasan failed to reach the primary endpoints in a Phase 2 clinical trial, possibly due to its induction of an alternative, proinflammatory form of cell death it induced [52, 53]. However, emricasan is well tolerated and was successful in a Phase 1 clinical trial, suggesting that it may be safe and less toxic than other candidate molecules, such as toxins [54]; however, its use is currently limited to oral administration. In addition, we observed that emricasan induces robust antigen-specific serum IgG_1_ and BALF IgA without significantly inducing antigen-specific serum IgE, which is responsible for type 1 allergic hypersensitivity [55]; this may explain the reduced incidence of post-injection side effects.

We conclude that necroptosis, a type of programmed cell death, is a biological mechanism for inducing optimal immunity that may be a promising target for the development of pulmonary mucosal vaccine adjuvants for inducing lung IgA. The caspase inhibitor emricasan may be a candidate molecule whose adjuvanticity is almost entirely dependent on RIPK1/RIPK3-mediated necroptosis.

## Supplementary information

Video S1. Emricasan induced necroptosis and IL-1α release from cultured alveolar macrophages (AMs) (Related to Fig. 2)

Supplementary Information

Original Data

## Data Availability

The datasets generated during and/or analyzed during the current study are available in the gene expression omnibus database (GEO) of the National Center for Biotechnology Information (accession no. GSE310184). All the other data supporting this study are available upon request.
