# Supplementary material for: Resident alveolar macrophages are highly susceptible to the induction of RIPK1/RIPK3-mediated necroptosis which promote efficient pulmonary immune response
Source: Cell Death Dis. 2025 Dec 22;16(1):919. doi: 10.1038/s41419-025-08372-8 (PMC12749839; doi:10.1038/s41419-025-08372-8)
Supplement: Supplementary file 2 — Supplementary Information [file 41419_2025_8372_MOESM2_ESM.pdf]

Figures S1–S12 and Video S1

Figure S1. Cultured alveolar macrophages (AM) differentiate into CD11+ SiglecF+ cells (Related to Figure 1).

Figure S2. Comparison of production of the proinflammatory cytokines in cultured AM treated with emricasan, STING ligand, or TLR ligands (Related to Figure 1).

Figure S3. Pan-caspase inhibitors induce cell death and IL-1α release in cultured alveolar macrophages (AMs) (Related to Figure 1).

Figure S4. The difference of C57BL/6J and C57BL/6N, or the sex in mice does not significantly influence the induction of cell death and IL-1α release in cultured AM treated with emricasan.

Figure S5. Cell death and the release of cytokines (IL-1α and TNF) occurred simultaneously in the cultured AM following stimulation with emricasan.

Figure S6. Newly established RIPK3 knockout mice completely lack RIPK3 in protein level.

Figure S7. Caspase inhibition-induced IL-1α release is associated with RIPK3-dependent necroptosis in cultured alveolar macrophages (AMs) (Related to Figure 2).

Figure S8. TNF signaling is dispensable in the necroptosis induced by emricasan. (Related to Figure 3).

Figure S9. Multiple caspase inhibition induced RIPK1/RIPK3-mediated necroptotic cell death in alveolar macrophages.

Figure S10. Emricasan is an intranasal mucosal adjuvant that enhances influenza virus-specific immune responses.

Figure S11 and S12. Gating strategy for flow cytometric analysis of BALF (Related to Figure 4).

Video S1. Emricasan induced necroptosis and IL-1α release from cultured alveolar macrophages (AMs) (Related to Figure 2).

The representative image is an example of LCI-S live cell images of in vitro-cultured alveolar macrophages isolated from the lungs of C57BL/6j WT mice cultured with emricasan (40 μM). Combined images of differential interference contrast microscope; DIC, IL-1α signal (cyan) and SYTOX (magenta) are shown. Scale bar: 20 μm.

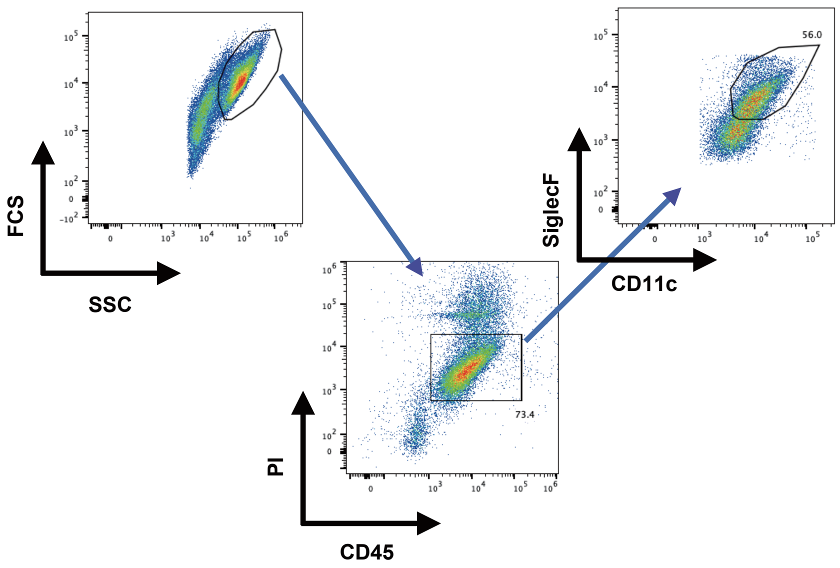

Supplemental figure 1. In vitro-cultured alveolar macrophages differentiated to CD11$^+$ SiglecF$^+$ cells. Representative data of the gating strategy for flow cytometric analysis of in vitro-cultured alveolar macrophages on day 6 of culture. The cells were collected and stained with anti-CD45-Pacific blue, anti-SiglecF-PE, anti-CD11c-Briliant Violet 421^{TM} and PI. Alveolar macrophages were defined as PI$^-$ CD45$^+$ CD11$^+$ SiglecF$^+$ cells (typical alveolar macrophages).

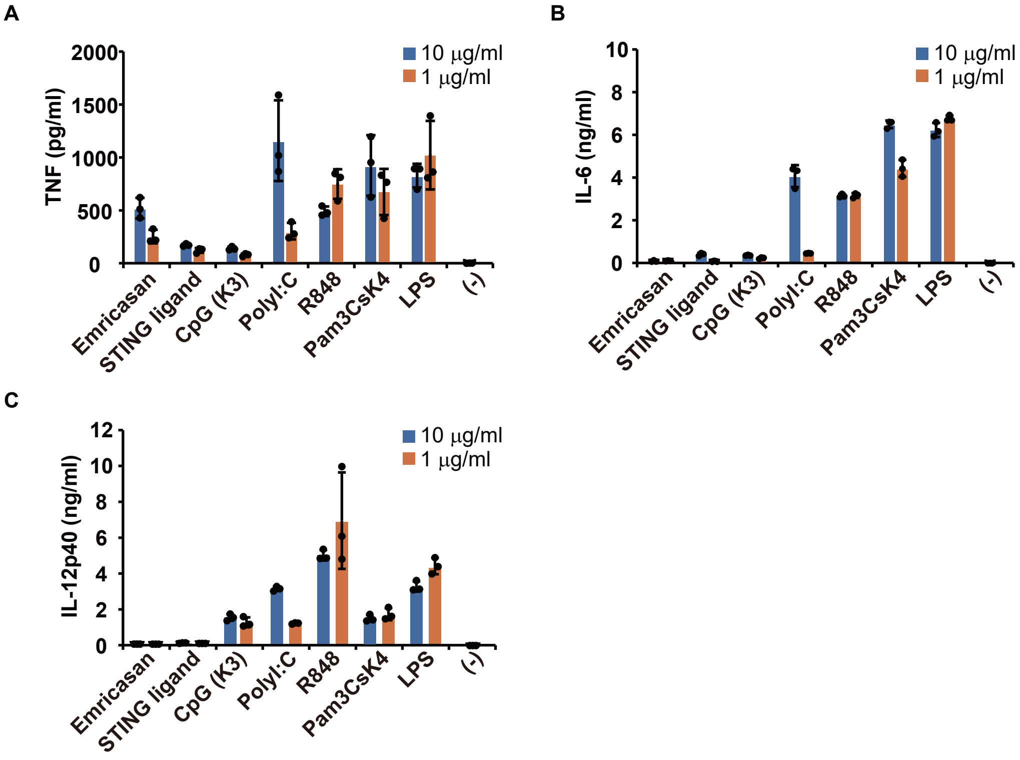

Supplemental figure 2. Pro-inflammatory cytokine production in cultured AM treated with emricasan, STING ligand or TLRs ligands.

(A-C) Cultured AM were cultured in the presence or absence of emricasan, STING ligand (2’ 3’ -cGAMP), CpG (K3), poly I:C, R848, Pam3CsK4, and LPS (1 or 10 μg/mL) for 12 hours. The culture supernatants were collected and measured inflammatory cytokines (TNF, IL-6 and IL-12p40) by ELISA. Data are representative of at least 2 independent experiments (n = 3/group).

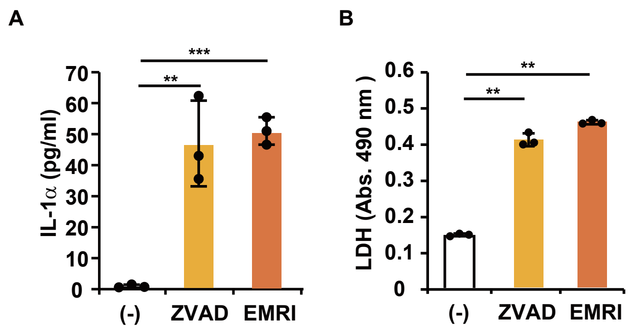

Supplemental figure 3. Pan-caspase inhibitors induce cell death and IL-1α release in cultured AM.

(A, B) In vitro-cultured alveolar macrophages isolated from the lungs of C57BL/6 mice were cultured in the presence or absent of emricasan (Emri, 40 μM) or zVAD-fmk (ZVAD, 20 μM) for 12 hours. IL-1α level of the culture supernatant was measured by ELISA (A). The cytotoxicity was measured by LDH assay (B). Data are representative of at least 2 independent experiments (n = 3/group). Error bars represent mean ± SD. *P* values are calculated by one-way ANOVA (Tukey's multiple comparisons test). **P*< 0.05, ***P*< 0.01, ****P*< 0.001 and n. s. (not significant).

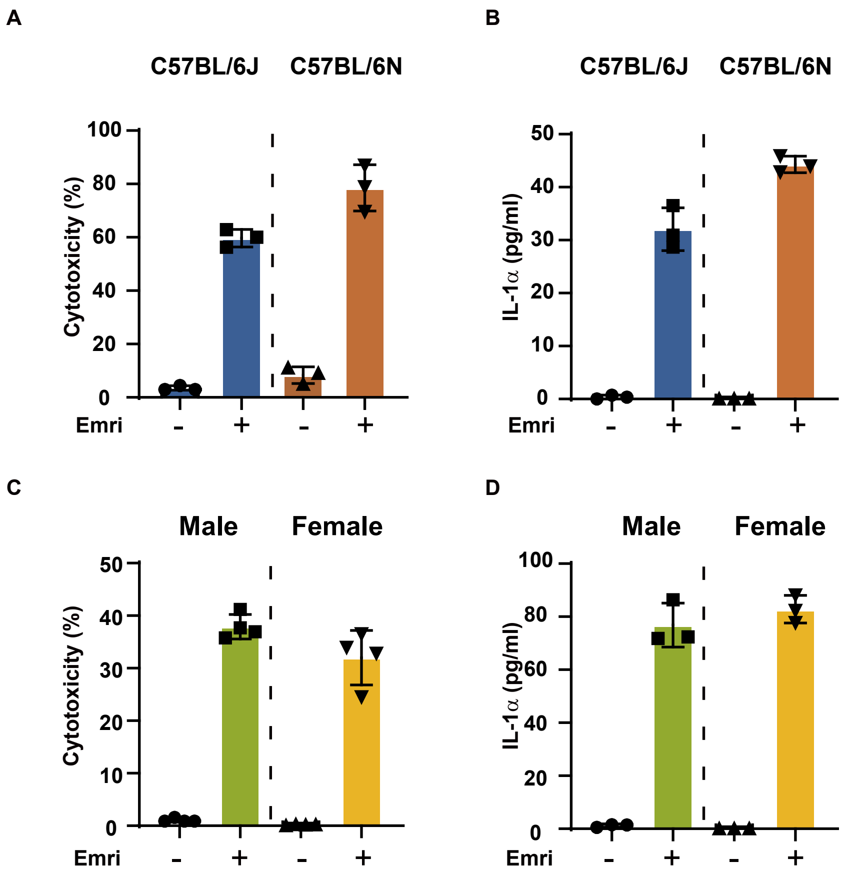

Supplemental figure 4. The difference of C57BL/6J and C57BL/6N, or the sex in mice does not significantly influence the induction of cell death and IL-1α release in cultured AM treated with emricasan.

(A, B) Cultured AMs of either C57BL/6J and C57BL/6N mice were treated with or without emricasan (40 μM) for 12 hours. Cytotoxicity was measured with LDH assays (A). IL-1α level of the culture supernatant were measured using ELISA (B).

(C, D) Cultured AMs of either C57BL/6J male and female mice were treated with or without emricasan (40 μM) for 12 hours. Cytotoxicity was measured with LDH assays (C). IL-1α level of the culture supernatant were measured using ELISA (D).

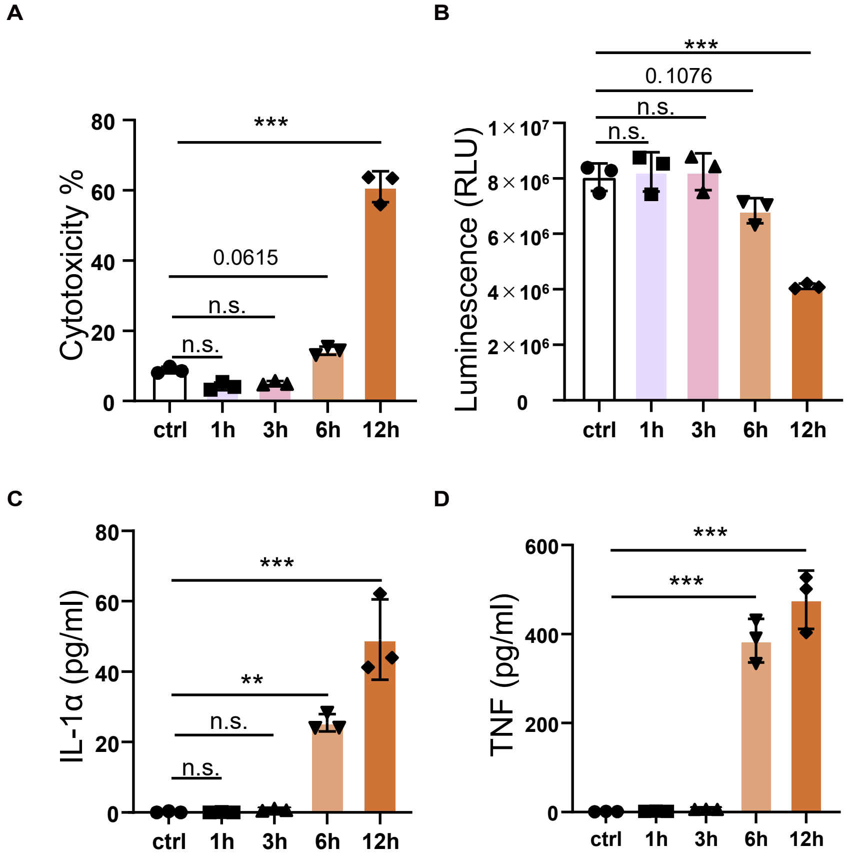

Supplemental figure 5. Cell death and the release of cytokines (IL-1α and TNF) occurred simultaneously in the cultured AM following stimulation with emricasan.

(A-D) Cultured AMs were treated with emricasan (40 μM) for 1, 3, 6 and 12 hours. Cytotoxicity and cell viability were measured using LDH assays and CellTiter-Glo assay (A, B). Cytokine levels (IL-1α and TNF) in the culture supernatant were measured using ELISA (C, D).

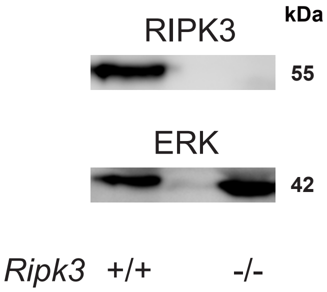

Supplemental figure 6. Newly established RIPK3 knockout mice completely lack RIPK3 at the protein level.
Peritoneal resident cells from *Ripk3*$^{+/+}$ (WT) and *Ripk3*$^{-/-}$ were isolated and the whole cell lysate ($5.0x10^5$) was used for the detection of RIPK3 at the protein level. The lysate was subjected to western blot analysis for the detection of RIPK3 and ERK (reference protein). The lysate was mixed with SDS loading buffer and subjected to glycine-SDS-PAGE (10% acrylamide separation gel) following transfer to PVDF membrane. Immunoblotting was performed by probing with anti-RIPK3 (1/1000) and anti-ERK (1/1000) antibodies diluted with 5% skim milk in TBST.

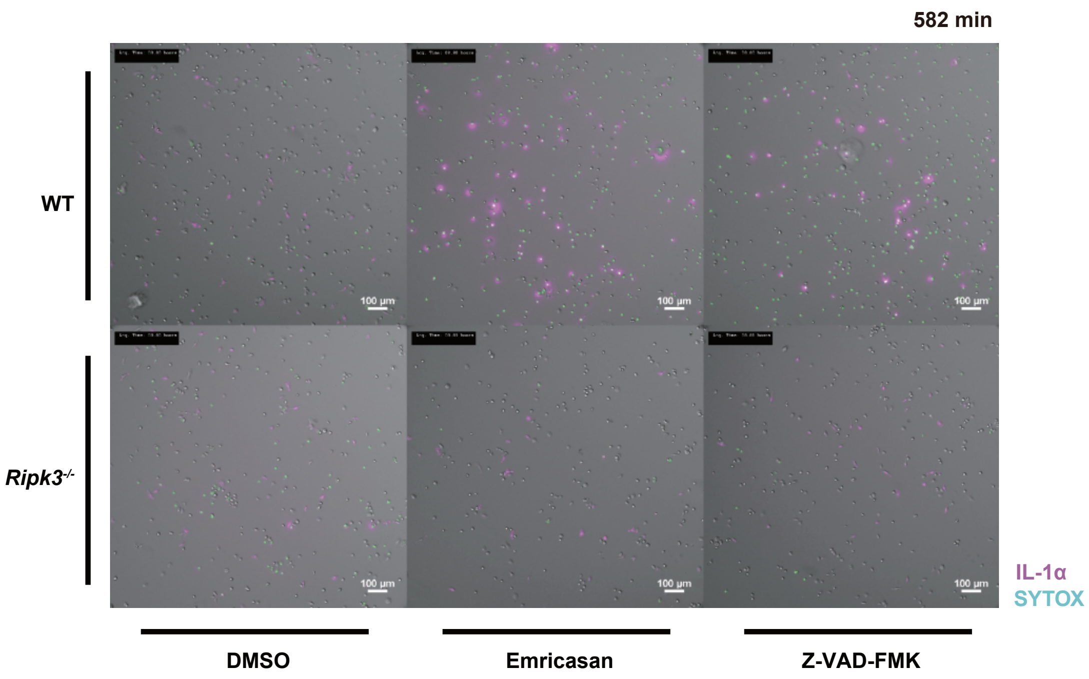

Supplemental figure 7. The caspase inhibition induced IL-1α release is associated with RIPK3-dependent necroptosis in cultured AM. Representative images are example of LCI-S live cell images of in vitro-cultured alveolar macrophages isolated from the lungs of C57BL/6 WT and *Ripk3*$^{-/-}$ mice cultured with DMSO (control, 0.1%), emricasan (40 μM) or zVAD-fmk (20 μM). Combined images of differential interference contrast microscope; DIC, IL-1α signal (magenta) and SYTOX (cyan) are shown. Scale bar: 100 μm.

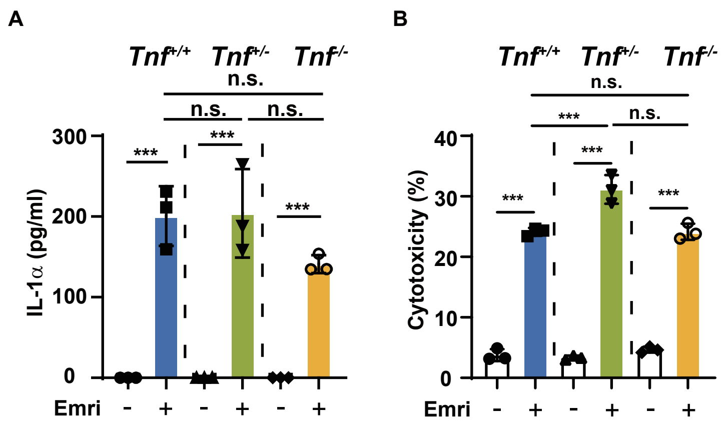

Supplemental figure 8. TNF signaling is dispensable in the necroptosis induced by emricasan.

(A, B) Cultured AM isolated from the lungs of *Tnf*$^{+/+}$, *Tnf*$^{+/-}$ and *Tnf*$^{-/-}$ C57BL/6J mice were cultured in the presence of emricasan (Emri, 40 μM) for 12 hours. IL-1α level of the culture supernatant was measured by ELISA (A). The cytotoxicity was measured by LDH assay (B). Data are representative of at least 2 independent experiments (n = 3/group). Error bars represent mean ± SD. *P* values are calculated by one-way ANOVA (Tukey's multiple comparisons test). **P*< 0.05, ***P*< 0.01, ****P*< 0.001 and n. s. (not significant).

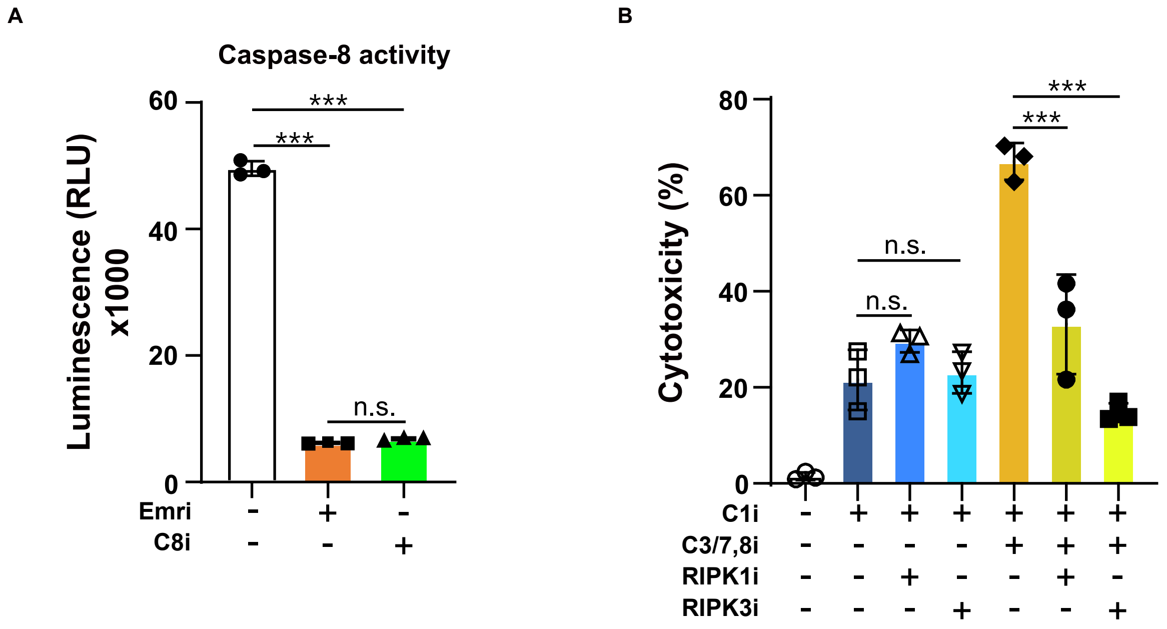

Supplemental figure 9. Multiple caspase inhibition induced RIPK1/RIPK3-mediated necroptotic cell death in alveolar macrophages.

(A) Cultured AM treated with emricasan (40 μM) or Z-IETD-FMK (C8i), (40 μM) were subjected to Caspase 8-Glo assay (n = 3/group).

(B) Cultured AM mice were pretreated with 10 μM of RIPK1 or RIPK3 inhibitors (RIPK1i: necrostatin-1, RIPK3i: GSK872) for 1 hour and then treated with 40 μM of caspase-1 inhibitor (C1i) or the mixture of caspase inhibitors C1i: caspase-1 inhibitor, C3/7i: caspase-3/7 inhibitor, C8i: caspase-8 inhibitor) for 12 hours. The cytotoxicity was measured by LDH assay. Error bars represent mean ± SD. P values were calculated by one-way ANOVA analysis of variance (Tukey's multiple comparisons test). *P< 0.05, **P<0.01, ***P< 0.001, n. s. (not significant).

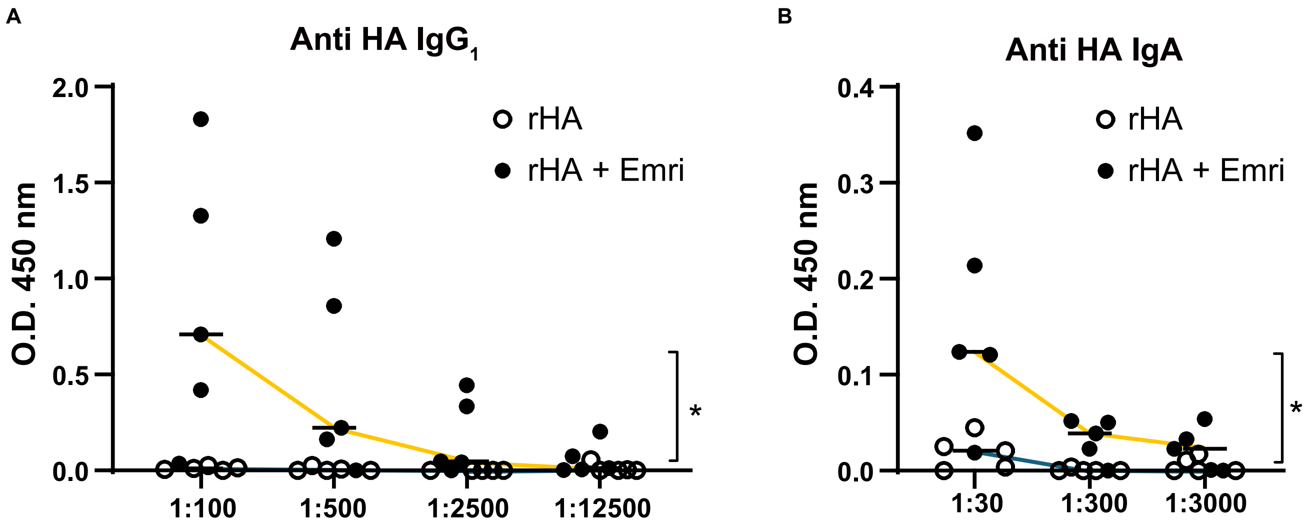

Supplemental figure 10. Emricasan is an intranasal mucosal adjuvant that enhances influenza virus-specific immune responses.

(A, B) C57BL/6J WT mice received recombinant H1N1 influenza virus (strain: A/California/07/09) HA antigen (rHA, 5 μg) with or without emricasan (Emri, 0.8 mg/kg) intranasally on days 0 and 10. On day 17, the mice were sacrificed and serum and BALF were collected. HA-specific IgG1 (A) (n = 5/group) and IgA (B) (n = 5/group) titers were analyzed using ELISA. P values were calculated by two-way ANOVA analysis of variance. **P*< 0.05, ***P*<0.01, ****P*< 0.001, n. s. (not significant).

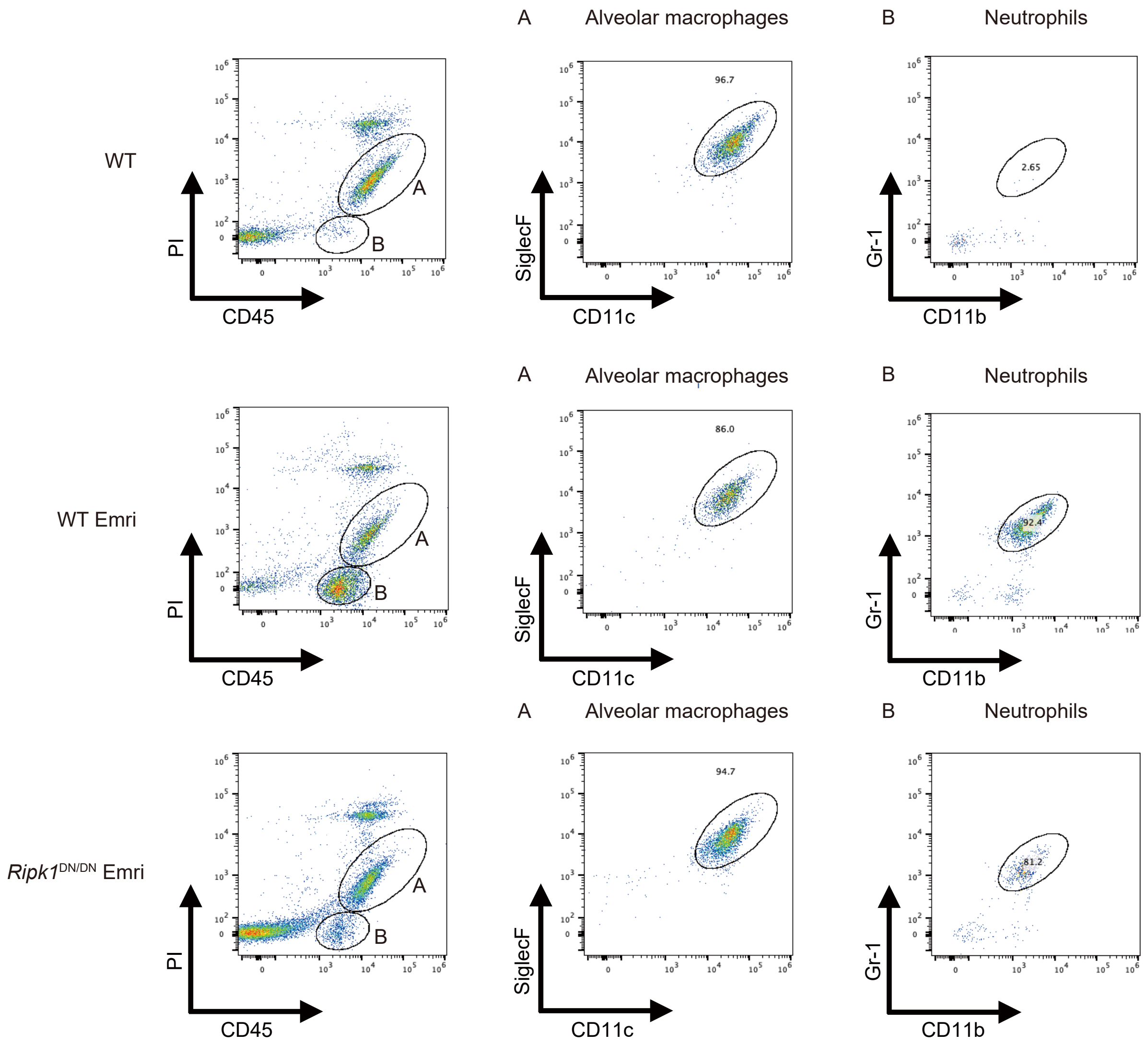

Supplemental figure 11. Gating strategy for flow cytometric analysis of BALF

Gating strategy for flow cytometric analysis of BALF after administration of emricasan (related to Figure 4C). C57BL/6j WT and *Ripk1*$^{DN/DN}$ mice were administered with emricasan (0.8kg/ml) in PBS intranasally. 10 hours after the administration, BAL fluid was collected by instillation of PBS 500 μl. BALF cells were gated as PI^{-}, CD45^{+} cells first and identified 2 discrete population as population A and B, respectively. Populations A and B were stained as CD11c^{+} and SiglecF^{+}, defined as typical alveolar macrophages, or CD11b^{+} and Gr1^{+}, defined as neutrophils. Data are representative of at least 2 independent experiments.

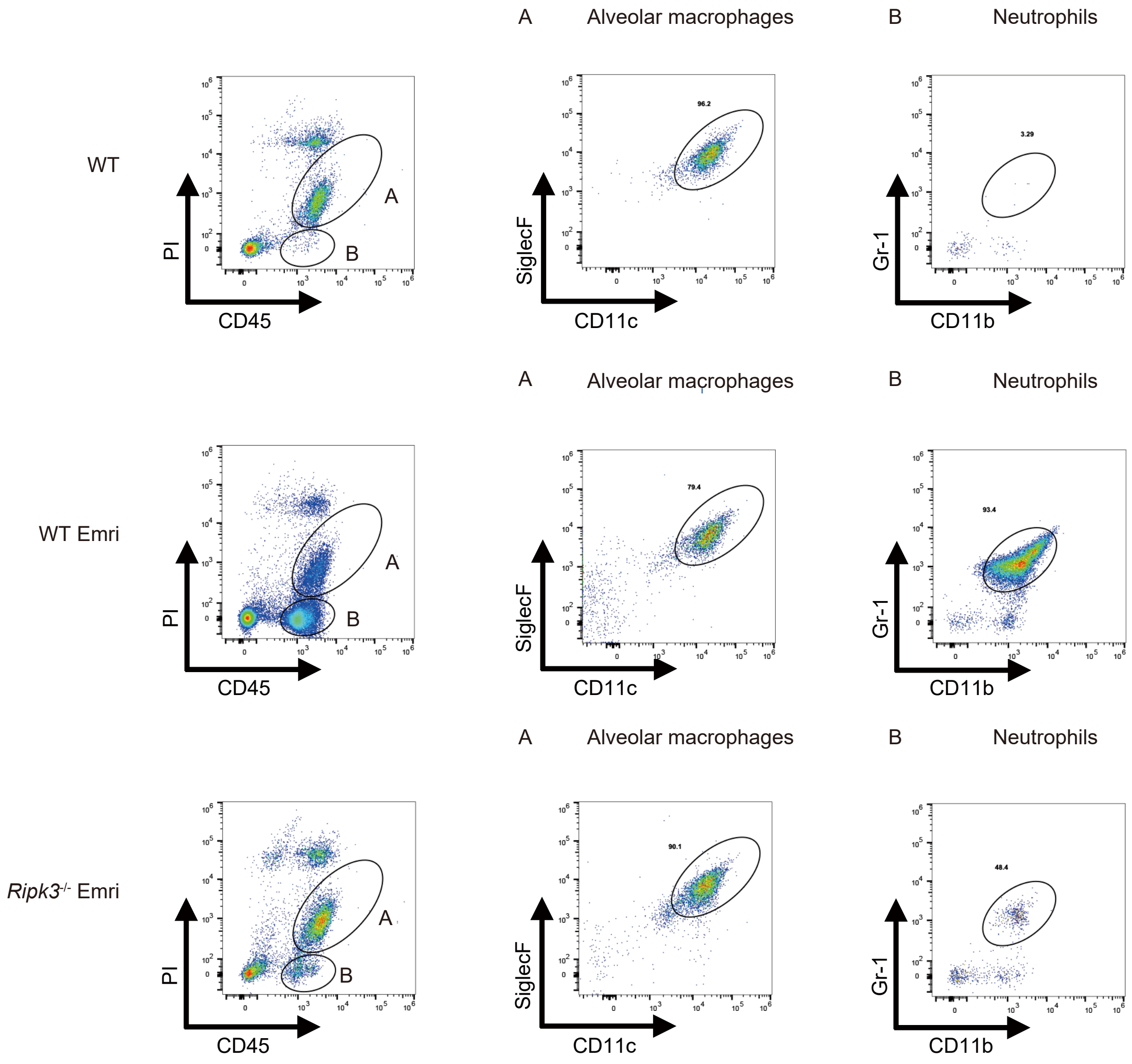

Supplemental figure 12. Gating strategy for flow cytometric analysis of BALF

Gating strategy for flow cytometric analysis of BALF after administration of emricasan (related to Figure 4D). C57BL/6j WT and *Ripk3*$^{-/-}$ mice were administered with emricasan (0.8 kg/mg) in PBS intranasally. 10 hours after the administration, BAL fluid was collected by instillation of PBS 500 μl. BALF cells were gated as PI^{-} CD45^{+} cells first. The cells were further gated as CD11c^{+} SiglecF^{+} population (A), defined as typical alveolar macrophages, and CD11c^{-} CD11b^{+} Gr1^{+} population (B), defined as neutrophils. Data are representative of at least 2 independent experiments.

Supplemental Table Reagents and Resources

REAGENT or RESOURCE	SOURCE	CATALOGUE NUMBER
Antibodies		
Anti-Cleaved Caspase-8 (Asp387)	Cell Signaling Technology	Cat# 8592
Anti-Caspase-8 Antibody	proteintech	Cat# 66093-1-Ig
Purified Mouse Anti-RIP Clone 38	BD Biosciences	Cat# 610458
RIPK3/RIP3 Antibody	Novus Biologicals	Cat# NBP1-77299
Anti-ERK antibody (C-14)	Santa cruz	Cat# sc-154
Purified Goat Anti-Mouse IgE	Southern biotech	Cat# 1110-01
Goat Anti-Mouse IgG_1-HRP	Southern biotech	Cat# 1070-05
Goat Anti-Mouse IgA-HRP	Southern biotech	Cat# 1040-05
Mouse anti OVA Monoclonal IgG_1 Antibody (L71)	Chondrex	Cat# 3008
Mouse anti OVA Monoclonal IgE Antibody (E-C1)	Chondrex	Cat# 3006
Mouse anti OVA Monoclonal IgA Antibody (2G12E12)	Chondrex	Cat# 7090
Goat anti rabbit IgG-HRP-conjugated	BIORAD	Cat# 170-6515
Goat Anti-Mouse IgG Polyclonal antibody, Horseradish peroxidase Conjugated	Millipore	Cat# AP124P
Rat Anti-Mouse Il-1 alpha / il-1f1 Monoclonal antibody (40508)	R&D Systems	Cat# MAB400
Goat Anti-Mouse Il-1 alpha / il-1f1 Polyclonal antibody, Biotin Conjugated	R&D Systems	Cat# BAF400
Anti-CD11c-Briliant Violet 421™ (N418)	BioLegend	Cat# 117343
Anti-SiglecF-PE (S17007L)	BioLegend	Cat# 155506
Anti-CD45-Pacific blue (30-F11)	BioLegend	Cat# 103126
Anti-CD11b-PE (M1/70)	BioLegend	Cat# 101208
Anti-Gr-1-PerCP-Cy5.5 (RB6-8C5)	BioLegend	Cat# 108428
Anti-CD16/32 (93)	BioLegend	Cat# 101302
Anti-CD11c biotin (N418)	BioLegend	Cat# 117304
Chemicals, peptides, and recombinant proteins		
HRP-conjugated OVA	BIORAD	Cat# BUF048
Emricasan	Cayman Chemical	Cat# 22204
zVAD-fmk	Peptide Institute	Cat# 3188-v
Belnacasan (VX-765)	Cayman Chemical	Cat# 28825
Z-IETD-FMK	Selleck	Cat# S7314
Ac-DEVD-CHO	Selleck	Cat# S7901
GSK872	Selleck	Cat# S8465
Necrostatin-1	Selleck	Cat# S8037
Recombinant mouse M-CSF	Peprotech	Cat# 315-02
Ovalbumin	FUJIFILM	Cat# 018-09882
ECL Prime Western Blotting Detection Reagent	Amersham	Cat# RPN2232
2.5 g/l-Trypsin / 1 mmol/l-EDTA Solution	Nacalai	Cat# 35554-64
Collagenase Type A	Wako	Cat# 034-24563
DNase I	Roche	Cat# 11284932001
Propidium iodide	Sigma Aldrich	Cat# 127616
CF®660R Streptavidin	Biotium	Cat# 29040
SYTOX™ Orange nucleic acid stain *5 mM solution in DMSO*	Thermo Fisher Scientific	Cat# S11368
Mineral oil	Merck	Cat# M5310
ReverTra Ace® qPCR RT Master Mix	TOYOBO	Cat# FSQ-201
RQ1 RNase-Free DNase	Promega	Cat# M6101
premix Ex Taq	Takara	Cat# RR039A
SYBR® Premix Ex Taq™	Takara	Cat# RR041A
TMB 2-Component Microwell Peroxidase Substrate Kit	Sera care	Cat# 5120-0050
Protease Inhibitor Cocktail	NACALAI TESQUE	Cat# 04080-24
iBlot™ 2 Transfer Stacks, PVDF, mini	Thermo fisher Scientific	Cat# IB24002
2'3'-cGAMP	InvivoGen	Cat# vac-nacga23
CpG ODN (K3)	GeneDesign	Cat# 40414605-001
Poly(I:C)	Amersham Biosciences	Cat #27-4732-01
R848	InvivoGen	Cat# tlrl-r848-1
Pam3CsK4	InvivoGen	Cat# tlrl-pms
LPS from *Escherichia coli* (O55:B5)	Sigma	Cat# L2880-10MG
Streptavidin MicroBeads	Miltenyi Biotec	Cat# 130-048-101
Alhydrogel® adjuvant 2%	InvivoGen	Cat# vac-alu-250
Recombinant Mouse IL-1α	R&D Systems	Cat# 400-ML-050/CF
Recombinant Mouse TNF	R&D Systems	Cat# 410-MT-050/CF
Recombiant H1N1 influenza virus HA protein (strain: A/California/07/09)	BIKEN Foundation	Not available
Commercial assay kit		
Caspase-8 Glo assay system	Promega	Cat# G8200
CellTiter Glo assay system	Promega	Cat# G7570
LDH Cytotoxicity Assay Kit	Promega	Cat# J2380
Endospecy® ES-50M	SEIKAGAKU CORPORATION	Cat# 020150
FastGene™ RNA Basic Kit	NIPPON Genetics	Cat# FG-80250
ELISA Max™ standard set Mouse IL-1α	Biolegend	Cat# 433401
Mouse IL-6 DuoSet ELISA	R&D Systems	Cat# DY406-05
ELISA Max™ standard set Mouse IL-12p40	Biolegend	Cat# 431601
ELISA Max™ standard set Mouse TNF	Biolegend	Cat# 430901
Oligonucleotides		
Ripk1 Fw: GAAGGCATGTGCTACTTACATGACA	Saeed et al [78].	Not available
Ripk1 Rv: TAATGTGAAAGTCACGATCAACGAG	Saeed et al [78].	Not available
Ripk3 Fw: AGAACTGAAGAAGCTGGAGTTTGTG	Saeed et al [78].	Not available
Ripk3 Rv: ATCTTGACTGCTACATCATGGTTCC	Saeed et al [78].	Not available
Ripk3 crRNA1 5-TAA TGC ACC CTC ACG GAC CC-3	In this paper	Not available
Ripk3 crRNA3 5-AAG AGA GAC TGG CTA TCG TG-3	In this paper	Not available
Taqman probes		
Il1a	Applied Biosystems	Mm00439620_m1
Actb	Applied Biosystems	NM_007393.1
Tnf	Applied Biosystems	Mm00443258_m1
