# Supplementary material for: Resident alveolar macrophages are highly susceptible to the induction of RIPK1/RIPK3-mediated necroptosis which promote efficient pulmonary immune response
Source: Cell Death Dis. 2025 Dec 22;16(1):919. doi: 10.1038/s41419-025-08372-8 (PMC12749839; doi:10.1038/s41419-025-08372-8)

Figure 1

A

ctrl	emri 10	sting ligand 10	cpg 10	polyic 10	r848 10	pam3csk4 10	lps 10	emri 1	sting ligand 1	cpg 1	polyic 1	r848 1	pam3csk4 1	lps 1
0.446346	898.9421	1.397288	0.059014	78.80326	10.90844	39.22968	65.85143	483.4111	0.446346	0.253181	8.014681	9.209838	16.1667	60.49905
0.638514	783.762	1.584527	0.829688	245.5105	8.468918	44.29052	98.25084	509.1662	0.253181	0.829688	7.24113	8.915868	11.98106	95.55917
0.253181	977.9566	1.584527	0.638514	175.0451	16.38316	93.31354	134.9339	520.0586	0.253181	0.059014	8.014681	8.618706	16.1667	107.6557

B

ctrl	emri 10	sting ligand 10	cpg 10	polyic 10	r848 10	pam3csk4 10	lps 10	emri 1	sting ligand 1	cpg 1	polyic 1	r848 1	pam3csk4 1	lps 1
3.766172	21.29154	3.460488	1.574622	5.362502	2.72856	3.277279	6.412398	31.07984	1.695803	1.271962	1.938368	2.363503	2.424304	5.239304
4.194836	21.81861	2.606807	1.695803	4.931604	2.485121	2.606807	4.378801	25.40446	1.574622	1.453508	1.756419	2.485121	2.545956	5.855966
4.931604	23.73825	2.789461	1.999051	4.624323	2.72856	3.766172	4.747185	35.41418	1.756419	1.514056	1.877702	3.888564	2.241952	6.660138

E

ctrl	emri 1h	emri 3h	emri 6h
0.0019	0.003721	0.022718	0.049721
0.004216	0.003961	0.013415	0.060371
0.005601	0.00491	0.008549	0.046391

F

ctrl	emri 1h	emri 3h	emri 6h
0.011518	0.017824	0.105112	0.123279
0.021344	0.033262	0.133972	0.230047
0.018326	0.043889	0.120742	0.14161

G

ctrl	emri
-0.65507	60.43605
-0.76026	66.30095
-1.18379	62.32758

H

ctrl	emri
14.47861	546.4389
15.34948	585.8058
15.60048	844.0236

I

ctrl	emri
3.259	27.972
4.308	17.967
3.04	17.783

J

ctrl	emri 0.2	emri 0.4	emri 0.8
0.335343	0.416773	0.746715	2.374646
0.353403	0.49862	0.682021	2.436389
0.453098	0.691247	1.466733	2.685793
0.236376			

K

ctrl	emri 0.2	emri 0.4	emri 0.8
18.62729	21.81893	23.8034	48.42666
17.80126	22.47472	23.58038	46.2288
16.18164	17.80126	29.10165	29.5773
12.8873			

Figure 2

B

ctrl	emri	emri + r3i 1	emri + r3i 10	emri + r1i 1	emri + r1i 10
2.38233	574.5755	16.93924848	2.23570038	27.78614352	2.98837622
2.132059	517.3498	121.1473055	2.14791968	22.14740622	3.16940832
2.316098	598.966	110.3883995	2.18133648	29.8788	2.85372342

C

ctrl	emri	emri + r3i 1	emri + r3i 10	emri + r1i 1	emri + r1i 10
7.351219	2392.682	1957.463	92.64019	267.1933	31.17748
7.16241	1594.987	1339.022	87.47288	155.024	38.4072
5.98984	1406.185	770.6731	95.27548	131.0657	19.82921

D

ctrl	emri	emri + r3i 1	emri + r3i 10	emri + r1i 1	emri + r1i 10
5.598826	105.5678	7.804493	4.626062	75.59455	22.05589
6.086947	88.47413	8.297828	7.312317	81.01173	22.845
5.111865	86.51264	6.8213	3.899531	78.77099	20.22479

E

wt ctrl	wt emri	RIPK1DN ctrl	RIPK1DN emri
0.759641	427.7119	0.817931	52.30295
0.817931	414.1403	0.606352	52.09047
0.65383	313.0677	0.58398	47.26815

F

wt ctrl	wt emri	RIPK1DN ctrl	RIPK1DN emri
4.216692	3071.235	4.216692	58.78313
4.729646	3217.495	4.729646	59.99461
1.998412	2795.396	1.998412	72.34405

G

wt ctrl	wt emri	RIPK1DN ctrl	RIPK1DN emri
13.94514	87.69581	10.13384	11.30619
16.61683	81.80183	8.34307	10.76429
16.15386	94.77588	7.986785	11.30619

H

wt ctrl	wt emri	RIPK3KO ctrl	RIPK3KO emri
0	360.1621	0	0
0	327.8993	0	0
0	347.849	0	0

I

wt ctrl	wt emri	RIPK3KO ctrl	RIPK3KO emri
0.526628	787.9062	0.803880012	89.30554044
1.080247	493.8678	0	85.23902352
0	545.6656	0.803880012	68.81189514

J

wt ctrl	wt emri	RIPK3KO ctrl	RIPK3KO emri
3.755	37.429	2.966	3.744
2.011	19.029	1.543	2.594
1.811	33.701	2.187	2.758

Figure 3

A

alv ctrl	alv emri	bmdm ctrl	bmdm emri
0.887	383.975	0.656	0.625
0.607	330.539	0.588	0.634
0.588	218.296	0.676	0.742

B

alv ctrl	alv emri	bmdm ctrl	bmdm emri
17.345	94.361	10.828	11.66
12.182	100	7.772	13.449
9.693	100	4.395	2.517

C

Alvmp ctrl	Alvmp emri	Fibro ctrl	Fibro emri
4.076649	110.8882	0	0.704036
4.880493	175.8078	0	0.792899
4.234766	151.7123	0	0.814901

D

Alvmp ctrl	Alvmp emri	Fibro ctrl	Fibro emri
5.677	40.506	2.29	5.956
5.291	44.963	1.594	6.194
5.611	34.785	1.757	4.741

F

alv	alv emri	bmdm	bmdm emri
29372	8013	28375	11906
24404	7767	27057	9502
25272	6218	28542	8693

G

	ripk1			ripk3		
alv ctrl	0.078021	0.055169	0.074842	0.007442	0.005563	0.006944
alv emri	0.0625	0.073302	0.070316	0.00564	0.00639	0.006045
bmdm ctrl	0.046071	0.072796	0.038208	0.004613	0.006615	0.003988
bmdm emri	0.026278	0.048361	0.01937	0.002668	0.006045	0.001575

I

ctrl	casp1 inhibitor	casp 3/7 inhibitor	casp 8 inhibitor	casp 1,3/7,8 inhibitors
1.966	12.005	0.96	2.982	31.1
0	8.135	2.327	3.268	42.8
0	7.377	0.246	1.437	47.73

J

ctrl	casp1 inhibitor	casp 3/7 inhibitor	casp 8 inhibitor	casp 1,3/7,8 inhibitors
1.164	40.935	2.196	0.008	34.31
0.866	55.129	2.223	0	43.429
0.415	41.4	1.943	0	38.594

Figure 4

A

WT pbs	WT emri	RIPK1DN emri
0.528339	74.22671	1.293779
0.62587	13.32002	0.852871
0.556095	67.05102	0.597893
0.418205	67.78117	0.542206

B

WT	WT Emri	RIPK3KO emri
0.0955	37.179	0.51525
0	68.72225	0.139
0.3665	84.6055	0.60675
0.2505	95.87025	1.38525
0.17813	71.59425	0.661563

C

wt ctrl	wt emri	RIPK1DN emri
2	13397	1442
8	14622	4001
5	20148	25
	14893	34

D

WT	WT emri	RIPK3KO emri
61	2648	196
30	6928	97
21	7240	57
	3138	1672

E

ova	ova + alum	ova + emri
33.13	165.58	334.53
10.72	332.87	132
34.97	534.14	215.72
2.98	351.64	123.26
34.82		

F

ova	ova + alum	ova + emri
1	28.7	42.65
0.2	79.15	13.25
4.35	398.65	21.35
0.25	314.95	4.2
0.05	0	36.8
0.15	778.5	36.8
0	26.4	4.5
0.1	587.3	75.9
0	133.2	6.55
0		6.95
		7.7
		1.2
		0.6

G

RIPK1DN/+ ova	RIPK1DN/+ ova+emri	RIPK1DN/DN +ova	RIPK1DN/DN +ova+emri
0.66	0.225	0	0
0.955	1502.4	0	0
0.59	65.395	0	0
0.11	11.555	0	0.035
0.27	34.675		0.165
	103.45		
	178.06		
	1.985		

H

RIPK1DN/+ ova	RIPK1DN/+ ova+emri	RIPK1DN/DN +ova	RIPK1DN/DN +ova+emri
0.253	0.6268	0.4671	0.1964
0.3994	15.3	0.0683	0.3318
0.5466	5.3861	0.3093	0.1621
0	2.4484	0.1275	0
0	5.9491		0
	16.7564		
	41.2		
	0		

I

wt +ova	wt +ova+emri	RIPK3KO +ova	RIP3KO +ova+emri
0	51.15	5.3	9.1
12.15	34.55	0	58.1
4.1	43.9	12.35	5.55
0	188.55	0	4.4
8.9	11.5	0	0
47.65	102.45	0	16.35
23.5	12	0	11.25
98.55	408.85	21.05	6.45
41.75	37.6	44.35	11.25
0	495.95		

J

wt +ova	wt +ova+emri	RIPK3KO +ova	RIP3KO +ova+emri
0	25.7	0	30.8
0	14.4	0	1.1
0	49.2	0	0
0	28.6	0	0
0	134.9	0	24.1
7	26.8	0	0
0	59.6	0	3.6
0	63.1	0	0
0	378.5	0	0
0	19.6	0	0

Figure 5

A

wt +ova	wt +ova+emri	IL1R1KO +ova+emri
0.5	1058.02	703.815
0.025	598.7	198.565
0.02	217.765	0.305
1.35	1207.9	159.49
0.55	137.29	54.13
0.555	268.145	0.865
0.44	779.99	52.065
0.365	301.69	0.495
	278.675	0.41
		0.12
		0.215

B

wt +ova	wt +ova+emri	IL1R1KO +ova+emri
2	210.6	30.2
1.8	1.8	11.2
1.8	7.7	2.1
1.8	415.6	9.5
0.4	5.1	13.1
0	34.9	0
0	5.1	0
0	15.9	0
	22.8	0
		0
		0

C

ova	ova+rIL-1a	ova+emri
0.0122	0.4155	1.21545
0.0142	0.24915	0.01315
0.0255	0.1497	0.24915
0.02405	0.46875	0.248
0.01225	0.47395	0.11935
0.0013	1.6887	0.67865
0.22535	0.703	0.22795
0.0014	7.7205	0.8797
0.0126	0.3726	0.98505
	0.28475	

D

ova	ova+rIL-1a	ova+emri
2.8	47.9	87.4
1.8	4.7	4.7
1.7	8.1	14.5
1.2	52.6	73.2
7.3	35.7	11.8
2.1	24.3	2.3
21.2	82.7	16.7
1.9	16.7	39.3
1.6	17.2	234.7
	9.6	

E

ova	ova+rIL-1a	ova+emri
44.274	215.322	297.225
0	711.102	74.586
326.952	340.35	71.061
0	159.393	78.636
6.786	158.664	256.017
0	284.157	85.386
6.795	137.817	38.97
57.342	1575	30.012
13.851	363.456	156.39
	291.183	

F

ova	ova+rIL-1a	ova+rIL-1a+rTNF
0.63	22.46	168.49
1.01	19.36	87.79
0.44	60.06	156.94
0.58	44.65	73.55
1.85	3.01	112.52

G

ova	ova+rIL-1a	ova+rIL-1a+rTNF
20.79	274.11	267.795
33.006	39.108	290.997
0	380.688	141.072
10.566	238.425	63.495
45.204	16.707	53.331

H

ova	ova+rIL-1a	ova+rIL-1a+rTNF
1.83	5.49	283.89
1.44	6.61	115.92
0.94	18.65	138.36
0.19	13.36	46.6
1.91	4.34	71.01

I

ova+IL-1a	ova+IL-1a+tnf
81.93791	629.1753
495.0394	301.687
157.767	1112.482
187.2706	1158.359
180.164	2109.842

J

ova+IL-1a	ova+IL-1a+tnf
0.020028	1.060102
0.169019	0.398355
0.04899	0.980776
0.056034	0.733916
0.259771	1.331496

Figure S2

A

ctrl	emri 10	sting ligand 10	cpg 10	polyic 10	r848 10	pam3csk4 10	lps 10	emri 1	sting ligand 1	cpg 1	polyic 1	r848 1	pam3csk4 1	lps 1
4.527	531.405	212.124	144.291	1080.285	565.932	1488.36	1116.237	268.362	107.817	105.963	326.703	732.585	529.992	1011.654
5.025	774.456	200.934	184.5	1269.948	593.391	774.456	1112.61	402.072	153.21	93.897	469.902	1055.25	958.326	1745.178
4.8	637.227	231.216	152.466	1985.052	673.752	1183.404	872.604	258.111	159.717	72.78	280.791	1011.654	1039.662	1079.649

B

ctrl	emri 10	sting ligand 10	cpg 10	polyic 10	r848 10	pam3csk4 10	lps 10	emri 1	sting ligand 1	cpg 1	polyic 1	r848 1	pam3csk4 1	lps 1
0	42.76	414.4	324.93	3504.6	3140.44	6620.82	6587.31	25.89	77.67	211.35	421.61	3188.25	4427.12	6953.93
2.98	103.49	458.37	322.32	4359.67	3264.49	6607.84	6165.41	34.33	69.92	220.41	409.17	3042.11	4062.38	6710.96
16.12	47.94	324.28	358.83	4430.78	3108.39	6311.61	5933.14	53.76	102.19	155.76	403.27	3256.18	4862.28	6710.96

C

ctrl	emri 10	sting ligand 10	cpg 10	polyic 10	r848 10	pam3csk4 10	lps 10	emri 1	sting ligand 1	cpg 1	polyic 1	r848 1	pam3csk4 1	lps 1
0	0	96.687	1565.901	3159.903	4961.316	1334.841	3076.947	0	0	1561.128	1264.659	4846.908	1549.95	4042.101
0	0	129.234	1387.026	3281.967	4940.925	1396.545	3118.317	0	0	1036.218	1286.973	10097.21	1401.429	4451.457
0	0	121.32	1760.928	3021.039	5468.667	1706.874	3600.558	0	0	1126.104	1210.194	6149.616	2054.907	4967.547

Figure S3

A

ctrl	zvad	emri
2.14869	62.25607	50.90083
1.242947	42.81072	55.35024
1.395166	35.38033	46.50857

B

ctrl	zvad	emri
0.146	0.429	0.465
0.149	0.401	0.456
0.142	0.396	0.455

Figure S4

A

C57BL/6J ctrl	C57BL/6J emri	C57BL/6N ctrl	C57BL/6N emri
2.971	56.262	11.075	87.026
2.982	60.003	4.918	78.92
4.482	62.837	9.034	69.739

B

C57BL/6J ctrl	C57BL/6J emri	C57BL/6N ctrl	C57BL/6N emri
0.724	36.529	0	44.003
0.362	30.931	0	45.957
0	28.684	0	42.911

C

male ctrl	male emri	female ctrl	female emri
0.882	35.78	0.197	33.973
1.605	41.195	0.22	32.853
0.888	37.697	0.144	24.571
0.9	36.939	0	36.557

D

male ctrl	male emri	female ctrl	female emri
0.563	71.816	0	77.807
1.42	86.391	0	82.407
1.514	72.334	0	88.195

Figure S5

A

ctrl	1h	3h	6h	12h
8.564	5.401	5.762	15.491	63.418
9.835	4.004	4.732	14.411	63.713
8.022	3.236	4.918	13.117	55.971

B

ctrl	1h	3h	6h	12h
7471752	7433192	7499504	6322416	4029713
8387362	8526810	8782428	7044015	4212718
8284461	8750873	8440884	7153380	4079942

C

ctrl	1h	3h	6h	12h
0.379	0.125	1.113	24.098	41.213
0	0	0.64	28.344	62.171
0	0	0.517	24	43.949

D

ctrl	1h	3h	6h	12h
1.91	2.269	6.409	431.162	527.271
1.309	1.972	5.884	390.84	501.235
0.861	1.635	5.845	333.973	403.295

Figure S8

A

wt ctrl	wt emri	tnf het ctrl	tnf het emri	tnf ko ctrl	tnf ko emri
0	159.348	0	158.742	0	134.836
0	211.369	0	188.379	0	134.539
0	230.973	0	264.964	0	153.963

B

wt ctrl	wt emri	tnf het ctrl	tnf het emri	tnf ko ctrl	tnf ko emri
3.142	24.351	3.004	33.589	4.19	23.822
3.241	24.652	3.679	28.859	5.163	23.019
4.892	23.408	3.318	31.008	4.622	25.614

Figure S9

A

ctrl	emri	casp8 inh
48886	6088	6583
48377	6118	6960
50581	6322	6910

B

ctrl	casp1i	casp1i-gsk 10uM	casp1i-nec-1 10uM	caspsi	caspsi-gsk 10uM	caspsi-nec-1 10uM
0.874	27.53	31.327	27.263	68.077	41.631	17.019
1.2	22.083	26.972	23.391	62.826	21.605	13.859
2.404	15.038	30.602	18.626	70.291	36.245	13.434

Figure S10

A

	HA					HA + emri				
100	0.014	0	0.011	0.026	0.005	0.709	1.83	1.327	0.419	0.036
500	0.025	0	0	0.007	0	0.222	1.207	0.857	0.163	0
2500	0	0	0	0	0	0.043	0.334	0.444	0.048	0
12500	0.055	0.004	0	0	0	0.013	0.074	0.202	0.006	0.002

B

	HA					HA + emri				
30	0.045	0.025	0.021	0.004	0	0.019	0.352	0.214	0.124	0.121
300	0.004	0	0	0	0	0	0.039	0.023	0.05	0.052
3000	0	0	0.011	0.017	0	0	0.001	0.023	0.033	0.054

Figure 3E

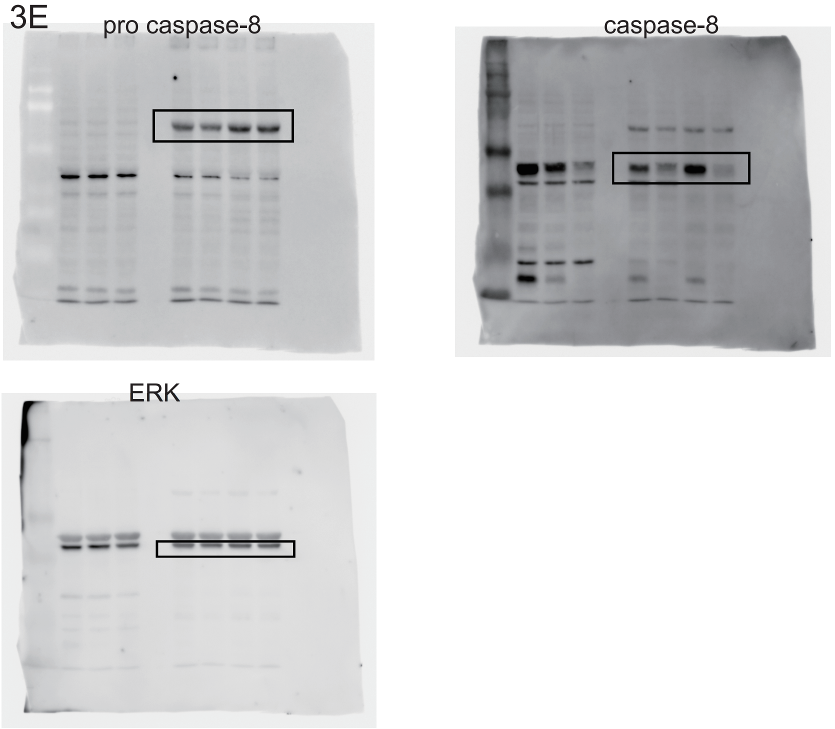

Figure 3H

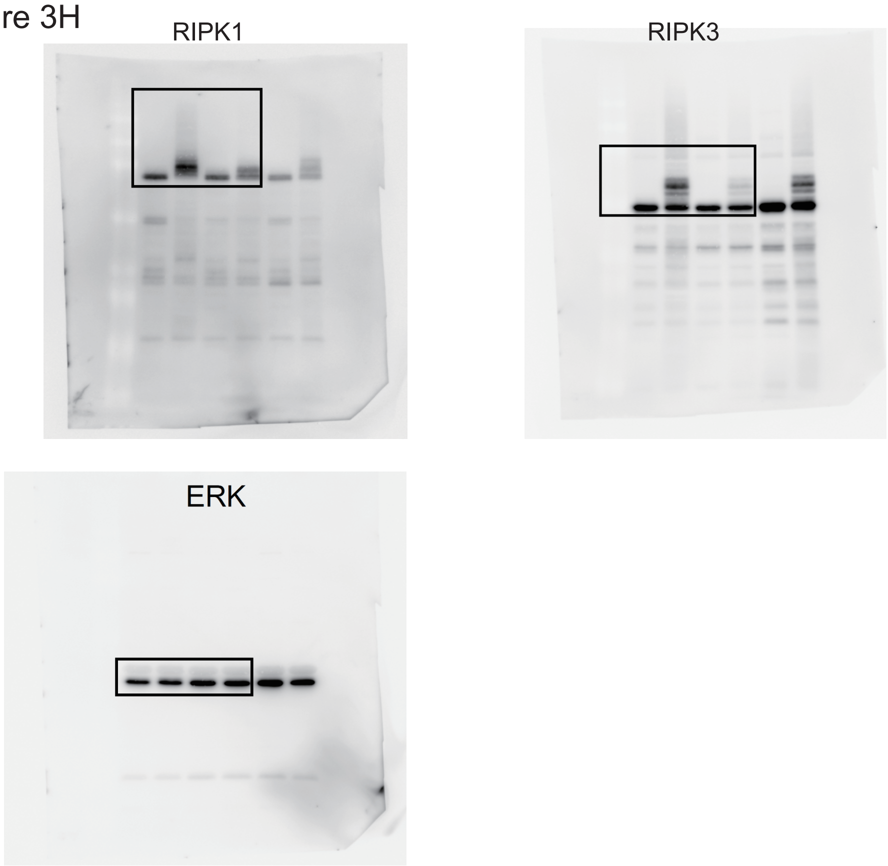

Figure S6

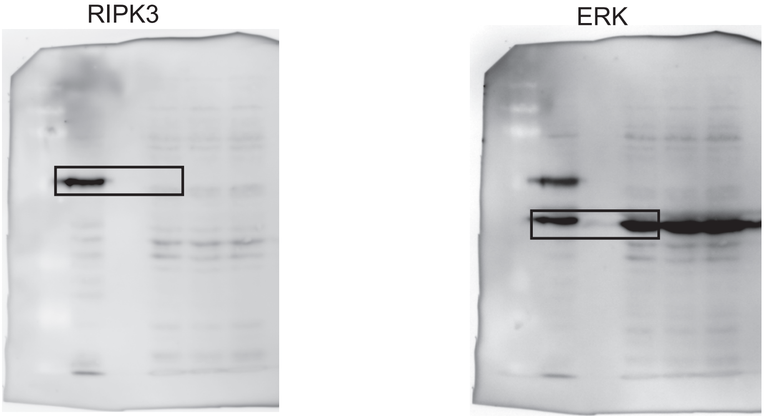

Supplement: Supplementary file 3 — Original Data [file 41419_2025_8372_MOESM3_ESM.pdf]
